# Immunotherapy for tuberculosis: emerging modalities, cross-disciplinary innovations, and roadmaps for drug-resistant disease

**DOI:** 10.3389/fimmu.2026.1873405

**Published:** 2026-07-09

**Authors:** Junchi Xu, JunHeng Shen, Sufang Chen, Jie Chen, Xuanmiao Liu, Fei Gao, Jianping Zhang

**Affiliations:** 1 Department of clinical laboratory, The Affiliated Infectious Diseases Hospital, Suzhou Medical College of Soochow University, Suzhou, Jiangsu, China; 2 Department of clinical laboratory, The Fifth People’s Hospital of Suzhou, Suzhou, Jiangsu, China; 3 Department of clinical laboratory, Suzhou Hospital of Traditional Chinese Medicinel, Suzhou, Jiangsu, China; 4 Department of clinical Laboratory, Children’s Hospital of Soochow University, Suzhou, Jiangsu, China; 5 Department of clinical Laboratory, Xi’an Daxing Hospital, Xi’an, Shaanxi, China; 6 Department of clinical Laboratory, The Affiliated Suzhou Hospital of Nanjing Medical University, Suzhou Municipal Hospital, Gusu School, Nanjing Medical University, Suzhou, Jiangsu, China; 7 Suzhou Key Laboratory of Intelligent Critical Illness Biomarkers Translational Research, Suzhou, Jiangsu, China

**Keywords:** clinical trials, drug-resistant, immunotherapy, tuberculosis, vaccine

## Abstract

**Rationale:**

The emergence of multidrug-resistant tuberculosis (MDR-TB) and limitations of current antibiotic therapies highlight the urgent need for novel treatment strategies. While immunotherapy has revolutionized cancer treatment, its potential in TB remains underexplored, with existing reviews focusing narrowly on vaccines and phage therapy. A comprehensive synthesis of TB immunotherapy advancements is critical to guide clinical translation.

**Content:**

Tuberculosis (TB) has been recognized as one of the earliest diseases treated with immunotherapy. The BCG vaccine played a crucial role in controlling TB epidemics for a long period. However, progress in TB immunotherapy stagnated due to the attenuation of BCG strains and the long-standing neglect in TB immunology research. In recent years, advances in TB and immunology research, including novel immunological theories such as trained immunity, alongside breakthroughs in various immunotherapies for cancer treatment, have provided new perspectives for TB treatment. This review summarizes the immune response to TB, including roles of macrophages, T cells, and nonclassical immune cells. It evaluates progress in cell therapy, antibody therapy, microbial therapy, vaccines, and cytokine therapy. Clinical trials, mechanisms, and challenges (e.g., immune exhaustion, antigen heterogeneity) of TB immunotherapy are analyzed, drawing parallels with cancer immunotherapy.

**Conclusions:**

Immunotherapy offers multifaceted strategies to overcome *Mtb* drug resistance and reduce side effects. Key directions include identifying novel antigens, optimizing immune checkpoint modulation, and combining therapies. Advances in single-cell sequencing and bioengineering will accelerate development, making immunotherapy a promising adjunct to conventional TB treatment.

## 1 Introduction

For drug-susceptible tuberculosis (DS-TB), the standard short-course chemotherapy regimen recommended by WHO is widely applied clinically, mainly composed of four first-line anti-tuberculosis drugs: isoniazid, rifampicin, ethambutol and pyrazinamide. The total treatment course lasts for 6 months, divided into an intensive phase and a continuation phase. For multidrug-resistant tuberculosis (MDR-TB), which is defined as tuberculosis resistant to both isoniazid and rifampicin, clinicians adopt long-term combined regimens based on second-line anti-tuberculosis agents, including fluoroquinolones, injectable aminoglycosides, thioamides and other auxiliary drugs.

However, conventional anti-tuberculosis treatments face prominent clinical limitations: long medication cycles, severe systemic toxicity such as liver and kidney damage, gastrointestinal reactions and neurological damage caused by long-term use of chemical drugs. More importantly, the continuous emergence of drug-resistant strains and the low treatment success rate of MDR-TB have become major obstacles to global tuberculosis(TB) control. These drawbacks make it urgent to develop novel adjuvant therapeutic strategies represented by immunotherapy to complement traditional chemotherapy (1). Tuberculosis is among the first diseases managed with immunotherapy. The BCG vaccine (2) and IL-2 (3) remain critical tools for TB prevention and treatment. Long-term stagnation in basic immunological research has hindered the development of TB immunotherapy. Meanwhile, the global spread of MDR-TB (4) and emerging resistance to new anti-tuberculosis drugs further demonstrate the great potential of immunotherapy for refractory TB (5). Accordingly, there is an urgent need to leverage proven paradigms from cancer immunotherapy, synthesize the evolving landscape of tuberculosis immunotherapy, and achieving rapid progress in tuberculosis immunotherapy. While recent reviews such as Lyu et al.have categorized broad TB immunotherapy modalities and highlighted ESAT-6/di-O-acyl-trehalose-driven T cell exhaustion, most prior overviews only superficially cover cell, antibody and probiotic therapies and lack integrated cross-modality analysis for drug-resistant TB (6). This study seeks to systematically synthesize recent advancements in tuberculosis immunotherapy, with particular emphasis on examining the current applications, prevailing challenges, and prospective directions of immune cell therapy, antibody therapy, and microbiome-based interventions in tuberculosis, thereby identifying novel therapeutic targets and innovative strategies for tuberculosis immunotherapy.

## 2 TB immunity

**Figure 1:**
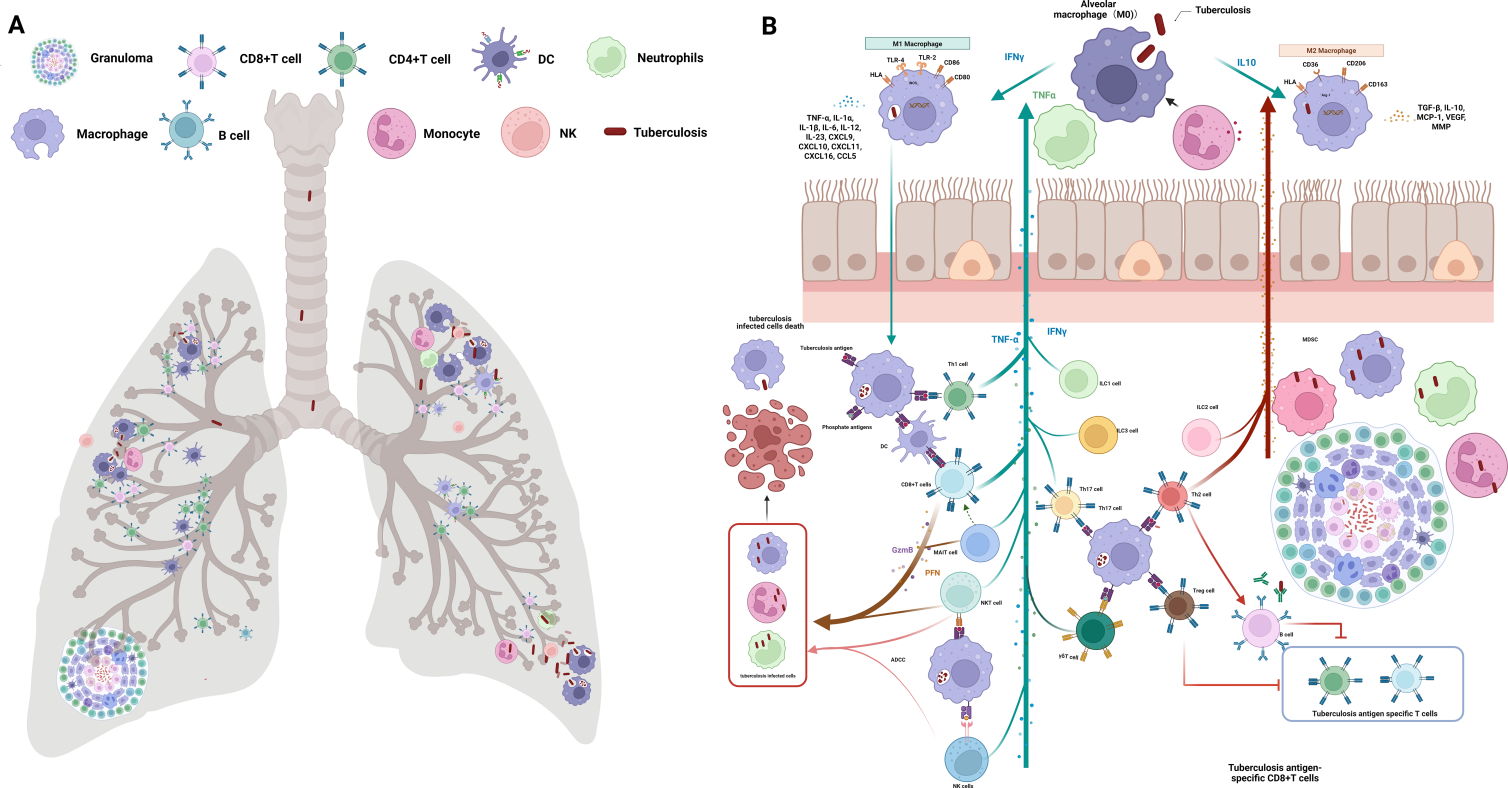
Immune network of the tuberculosis microenvironment. **(A)** Immunopathology of tuberculosis. **(B)** Tuberculosis immune microenvironment.

*Mycobacterium tuberculosis* (*Mtb*) is a pathogenic bacterium and the causative agent of TB, with the macrophage–T-cell axis serving as the primary host immune pathway for combating *Mtb* infection. Upon entry into the airways, *Mtb* is initially phagocytosed by pulmonary innate immune cells, including macrophages, dendritic cells (DCs), monocytes, and neutrophils, which then form phagosomes that undergo acidification to eliminate *Mtb*(**Figure 1A**) (7). Subsequently, the dead *Mtb* cells are phagocytosed by antigen-presenting cells (APCs), which process and degrade these pathogens into antigenic peptide fragments. These peptides are then loaded onto major histocompatibility complex (MHC) class II molecules on the APC surface, a process that subsequently triggers activation of adaptive immune cells—primarily CD4+ T lymphocytes—to secrete proinflammatory cytokines, including interferon-γ (IFN-γ) and tumor necrosis factor-α (TNF-α) (7). Upon entering alveolar macrophages, certain *Mtb* strains disrupt multiple host defense pathways to establish intracellular persistence: they impair phagolysosome biogenesis and maturation (8), disrupt the assembly of MHC class I-β2-microglobulin (β2M) antigen-presenting complexes on the macrophage surface, reduce responsiveness to IFN-γ (9), suppress autophagy (10) and downregulate stress adaptation gene expression (8, 11, 12). These coordinated perturbations collectively impair macrophage phagocytic function and antimicrobial activity. Following intracellular proliferation, Mtb disseminates to adjacent macrophages and dendritic cells (DCs). While antigen presentation by infected cells activates antigen-specific CD4^+^ and CD8^+^ T cells, Mtb concomitantly dysregulates T cell effector function, leading to impaired cytotoxicity and memory differentiation (13). Concurrently, infected cells secrete chemokines and inflammatory cytokines that recruit monocytes and neutrophils to the infection foci, driving the formation of tuberculosis granulomas (14) (**Figure 1B**).

T-cell-mediated cellular immunity is another critical component of the host defense against TB infection and plays an important role in mediating lung inflammation, inducing neutrophil infiltration, and initiating type I immune responses (15). The absence of CD4^+^ T cells results in a reduced ability of the host to control Mtb replication and leads to rapid cell death (16). Th1 cells can induce macrophage activation and polarization toward the M1 type, thereby inhibiting intracellular Mtb replication. Moreover, Th2 cells mediate humoral immunity against TB by secreting cytokines such as IL-4, but Mtb can suppress type I immune responses by altering the Th1/Th2 balance (17). Th17-induced neutrophil inflammation is a significant cause of tissue damage in TB, while regulatory T (Treg) cells reduce inflammation and tissue damage through the release of TGF-β (16). However, Mtb bacteria also use Treg cells to suppress host immunity against TB infection (18). CD8^+^ T cells also play an essential role in TB infection, as they can increase the production of IL-2 and IFN-γ after the bacteria binds to the natural killer cell receptors NKG2A and NKG2C, increasing the Mtb killing ability of macrophages (19). In chronic infection models, the exhaustion of CD8^+^ T cells impairs the long-term control of TB and the effectiveness of vaccines (20, 21). In *in vitro* cell models, the killing effect of cytotoxic lymphocytes (CTLs) on Mtb-infected cells was observed.

In addition to the core role of macrophages and T cells, a variety of other immune cells also participate in TB immunity. Neutrophils are involved in early Mtb infection (22), and mitochondrial metabolism in neutrophils increases in the lung tissue during TB infection, enabling neutrophils to selectively kill Mtb in cells by regulating lipid uptake (22, 23). The recruitment of monocytes to infection sites and their mediation of inflammation are critical components of innate immunity against TB (24). CD16+ monocytes recognize Mtb bacteria via CCR2, differentiate into macrophages and DCs under the stimulation of TB antigens, and participate in TB immunity through phagocytosis, oxidation, or adaptive immune responses (25). Mtb cell wall components can directly bind to NKp44, enabling NK cells to recognize Mtb-infected macrophages and directly kill them through antibody-dependent cell-mediated cytotoxicity (ADCC). NK cells also produce cytokines such as IL-22 to inhibit Mtb replication and enhance the ability of macrophages to kill Mtb (26). Moreover, a previous study revealed that TB antibodies regulate the fusion of phagolysosomes to promote the phagocytosis of Mtb by macrophages (27), and recruit follicular helper T cells to lymphatic follicles to control Mtb replication (28). Additionally, the mortality of TB-infected mice after B-cell knockout significantly increased (29). Some studies suggest that the number of TB antigens on the surface of DCs, the specificity of antigens for PRRs and the differences in PRR density, rather than the number of DCs, are key to inducing adaptive immune response (30). The autophagy marker protein LC3 and the autophagy adapter protein p62/SQSTM1 (p62) are highly expressed on the surface of Mtb-infected DCs, indicating that DCs may participate in TB immunity by inducing macrophage autophagy (31) and enhancing antigen presentation.

MAIT cells bind to MR1 ligands through Toll-like receptor (TLR)2/6 agonist and Pam2Cys (P2C) (32), and MAIT cells participate in TB immune responses by secreting IFN-γ, GZMB, and TNF after IL-23 stimulation (33). MAIT cells play a role in the early stages of TB infection through MR1 binding, but MAIT cells in the peripheral blood of patients with active TB decrease in number (34) and exhibit functional exhaustion. During Mtb infection, γδT cells are rapidly activated after stimulation by a phosphoantigen or heat-resistant antigen (Mtb-HAg) (35), releasing TNF-α, IL-17, and IFN-γ, which, along with cytotoxic molecules, reduce the activity of intracellular and extracellular Mtb (36). Mtb infection alters the phenotype and function of IL-18Rα+ ILCs in lung tissue, polarizing them into the IFN-γ-secreting ILC1 subset (37). In early TB infection, IL-23 upregulates the expression of CXCR5 on ILC3s, resulting in their accumulation in the lungs and increased secretion of IL-22 and IL-17, which exert anti-TB effects (38, 39). Mtb antigen-specific NKT cells exert their anti-TB effects by secreting cytokines and killing infected cells through ADCC. A previous study found that TB antigen-specific NKT cells participate in humoral immunity against TB by promoting immunoglobulin production by B cells through the upregulation of IL-21 expression.

The discovery of nonclassical immune responses (40) and the elucidation of the roles of various immune cells, such as B cells, ILCs, and NKT cellsin combatting TB had led to the identification of more targets for immunotherapy (41). Moreover, the functions of negative regulatory cells such as myeloid-derived suppressor cells (MDSCs) (42) and Treg cells in inhibiting immune responses in the context of lung injury and repair offer new research directions for TB treatment. Granulomas were previously considered immune-privileged sites, but a recent study has found that CD4^+^T cells rewire granuloma cellularity and regulatory networks to promote immunomodulation following Mtb reinfection (15), providing an new approach for TB prevention. Therefore, targeting key factors in the immune regulatory network and, through bioengineering and other means, enhancing the functions of lymphocytes that recognize TB antigens, inducing macrophage polarization toward the M1 phenotype, and balancing type I/II immune responses are new strategies for TB prevention, treatment, relapse, and repair.

## 3 Immune cell therapy and TB

Adoptive cell therapy has undergone five stages, each of which has its unique characteristics in terms of the methods used for culturing cells and clinical outcomes (**Supplementary Table 1**). Several animal studies have shown that the adoptive transfer of Vγ2Vδ2 T cells activated by BCG (43), phosphoantigen/IL-2, or attenuated HMBPP-producing *Listeria monocytogenes* (Lm*ΔactA* prfA*) can prevent Mtb infection in macaques, significantly reduce the Mtb load in lung tissue, and attenuate TB lesions (44). Human experiments have also revealed that adoptive therapy with Vγ2Vδ2 T cells can reduce the number of lung lesions and sputum count (45). Tuberculosis antigen-specific T cells are considered to be related to the prognosis of tuberculosis and are often used as an indicator to evaluate vaccine immunogenicity. The TB antigen-loaded DCs were used to treat TB-infected mice and found that the bacterial loads in the lungs and spleens of the mice were significantly reduced, along with a marked amelioration in lung pathology (46). Tang et al. conducted a clinical study on the use of cytokine-induced killer (CIK) cells to treat MDR-TB and reported that CIK cell immunotherapy combined with anti-TB chemotherapy increased sputum smear conversion rates, relieved symptoms, ameliorated lesions, increased recovery rates, and exhibited good safety (47). Paterson RL et al. engineered T cells by combining HLA-E with peptides encoded by the Mtb inhA gene, demonstrating that this approach could effectively eliminate Mtb-infected cells and inhibit intracellular Mtb replication (48).

**Figure 2:**
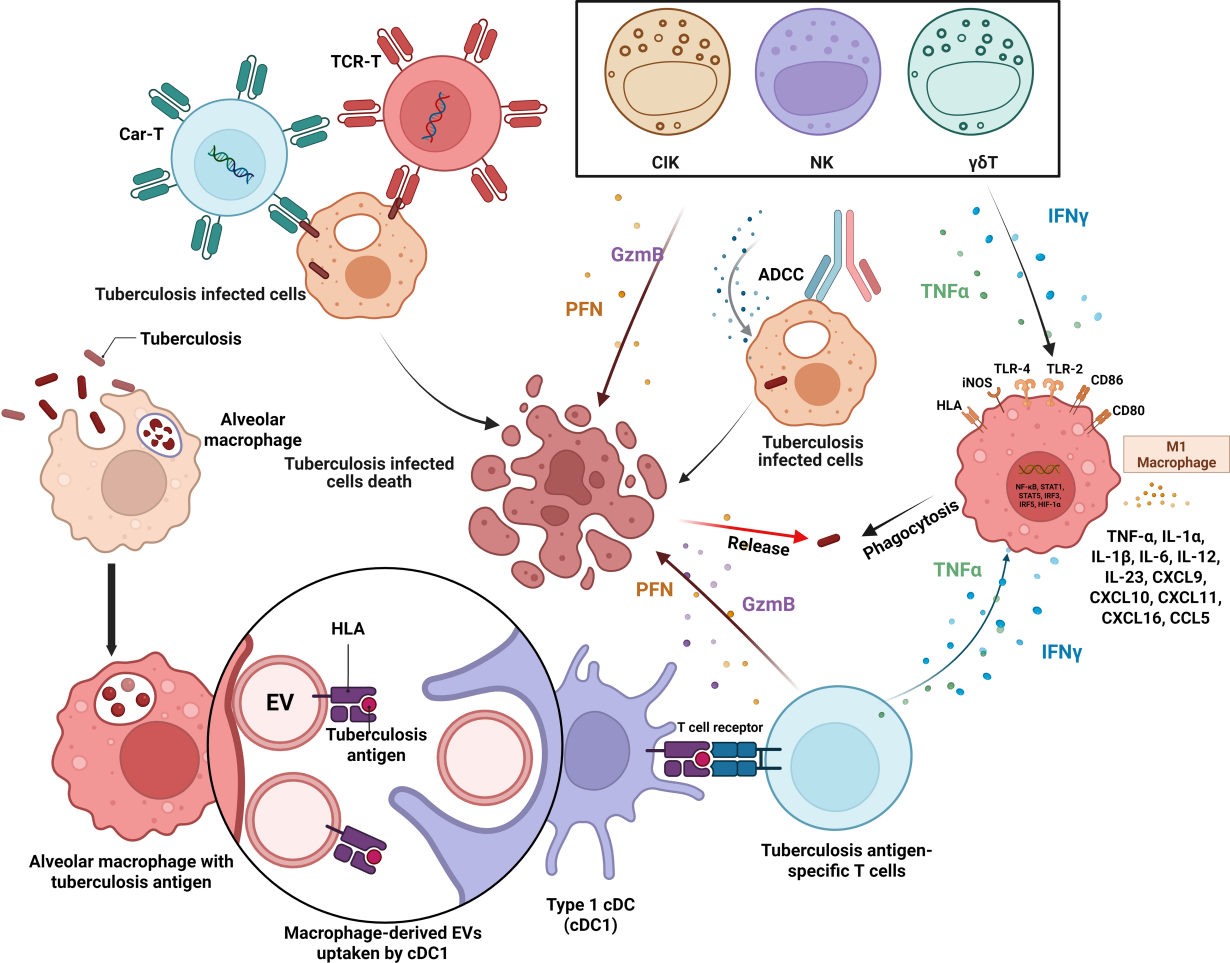
Mechanisms and development directions of immune cell therapy for tuberculosis.

The application of immune cell therapy in TB is still limited, with only four clinical trials(ChiCTR-INR-17012369, NCT05493267, NCT03575299, and ChiCTR-INR-16009606, **Supplementary Table 1**) currently underway. Drawing on the successful models of cancer immune cell therapy will provide insights for the clinical application of immune cell therapy for TB(**Figure 2**). Given that immunosuppression is more pronounced in cancer than in TB, and and TB antigens—being exogenous—are more readily captured and presented by APC, personalized immunotherapy regimens tailored to different TB stages may confer therapeutic benefits. Patients with early-stage TB infection have intact immune function and respond well to chemotherapy. The goal of cell therapy is to improve the quality of life of patients and shorten the course of treatment. Therefore, safe and simple cell therapy techniques such as CIK cell immunotherapy combined with chemotherapy better align with this goal. For patients with MDR-TB or other refractory TB, who have a prolonged disease course and poor baseline function, highly specific and functionally strong cell therapies such as CAR-T-cell therapy may be better treatment options, as long-term exposure of immune cells to high concentrations of TB antigens leads to immune exhaustion.

Despite the promising preclinical and preliminary clinical results, adoptive cell therapy for TB still faces many practical problems. First, the preparation process of CAR-T, γδT cells and other cell products is complex, with high technical requirements and expensive costs, which is difficult for large-scale clinical promotion in tuberculosis high-burden areas. Second, long-term exposure to Mtb antigens easily induces persistent immune exhaustion of infused immune cells, leading to decreased *in vivo* efficacy. Third, individualized cell preparation schemes lack unified industry standards, and there are potential risks such as off-target immune response and mild immune-related inflammatory adverse reactions in a small number of patients. In addition, the core TB-specific TCR/BCR clonotypes have not been fully clarified, which restricts the optimization of cell targeting ability.

Similar to cancer cell therapy, finding target antigens,optimizing CAR structures (49), and developing new technologies (such as CAR-NK (50) and CAR-macrophage (51)) are the development directions for TB cell therapy, in addition to optimizing combination immunotherapy and chemotherapy treatments. Previous studies have revealed diversity in the T-cell receptors (TCRs) of TB antigen-specific T cells (52) and strong resistance of NKs (53–55), ILCs (38, 56) and MAIT (34, 57, 58) to TB infection, and these cells may be new targets for TB cell therapy (59). Although cell therapy has enormous application potential in TB treatment, the TCR subgroups that primarily function in host defense against TB infection and their corresponding antigenic peptides are unclear, and a simple and efficient strategy to activate antigen-specific T cells remains to be further developed. Therefore, single-cell sequencing (60) and other techniques should be used to further explore the immune cell profiles during TB infection and provide reference data to resolve these issues in the future.

## 4 Antibody therapy and TB

**Figure 3:**
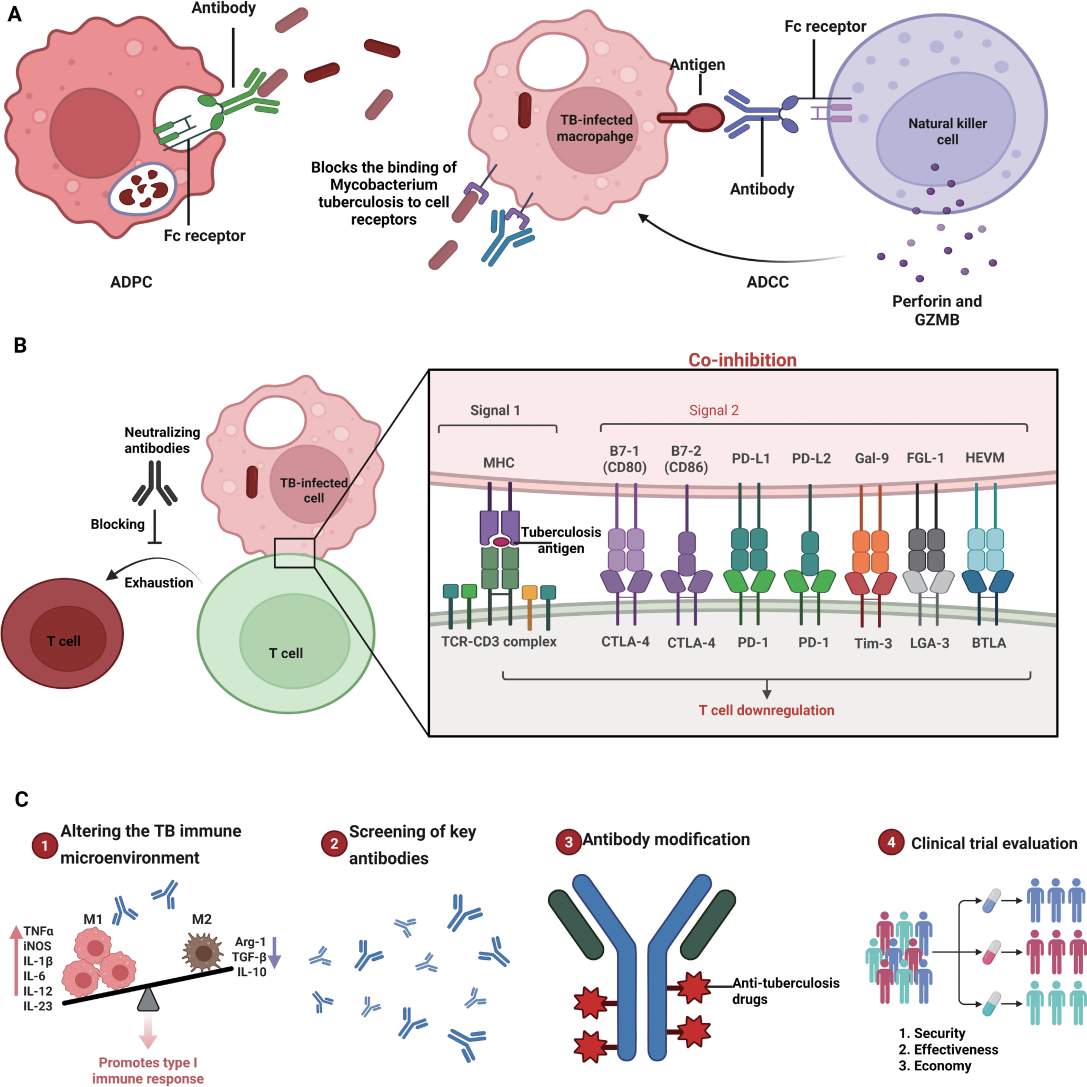
Mechanisms and development directions of antibody therapy for tuberculosis. **(A)** Mechanisms of antibody therapy for tuberculosis. **(B)** Mechanisms of immune checkpoint therapy for tuberculosis. **(C)** Development directions of antibody therapy for tuberculosis.

Studies have demonstrated that membrane protein (OmpA) monoclonal antibody can significantly reduce the bacterial load in the lungs and relieve symptoms (61–64), furthermore, the humoral immunity-targeting subunit vaccine Ag85A-LpqH confers robust protection against tuberculosis in mice (65). Enhancing phagocytosis, mediating ADCC effects, and blocking signaling pathways are the main mechanisms by which antibodies exert their effects. It has been demonstrated that antibodies can enhance Mtb phagocytosis (ADCP) (66), mediate ADCC, and inhibit Mtb infection of epithelial cells (27). Emerging evidence indicates that antibody-mediated restriction of Mtb involves reorganization of tissue-level immune responses to infection, requiring coordinated engagement of antibody Fab and Fc domains (67). Specifically, pre-existing mucosal (pulmonary) and systemic IgA targeting conserved Mtb glycan motifs confers protective immunity against TB. Mtb-specific antibodies also exert direct antimicrobial effects: they block Mtb adhesion to host cell receptors, preventing epithelial cell invasion (68), initiating ubiquitination-dependent bacterial degradation pathways. While early post-infection IgG directed against Mtb protein antigens provides an additional layer of defense (69) (**Figure 3A**).

Beyond antibody-mediated mechanisms, natural killer (NK) cells contribute to Mtb clearance by binding Mtb antigens via Fcγ receptors (FcγRs), thereby triggering ADCC against infected cells (66, 70). Additionally, antibodies isolated from latently infected individuals enhance phagolysosome maturation and induce non-pyroptotic inflammasome activation in macrophages, further augmenting antimicrobial activity (66) (**Figure 3A**).

Because of their characteristics, monoclonal antibodies are considered important tools for achieving clinical antibody therapy. With the isolation and identification of TB antigens, a variety of TB antigen-specific monoclonal antibodies have been developed and characterized (71). Irvine et al. found that the BCG vaccine can increase the antibody titer and that TB antigen-specific IgM monoclonal antibodies can reduce the survival rate of Mtb *in vitro (*72). Animal model studies have shown that alpha-crystallin (Acr/HspX) and PstS1 monoclonal antibodies can effectively reduce the load of Mtb in animal models (73, 74), while combination of monoclonal IgA 2E9 antibody against Acr/HspX antigen and IFN-γ to prevent multidrug-resistant *Mtb* (MDR-TB) infection (75). In the study by Fischinger et al., it was found that the Mtb antigen-specific IgG3 titer was reduced in recurrent TB patients (76). Huoming LiI et al. identified a protective antibody targeting the outer membrane protein of *Mtb* and can reduce the organs’ bacterial burdens and pathological damages in the prevention mouse model (61). In addition to TB antigen-specific antibodies, other TB infection-related antibodies, such as human IgG1 P1AM25 has demonstrated protective efficacy in passive transfer experiments using Mtb-infected FcγR-humanized mice (77).

Studies have found that TB infection can also lead to the abnormal expression of immune checkpoint molecules (78), inducing changes in T-cell function (79)(**Figure 3B**, **sTable 2**). After Mtb infection, the levels of PD-1 and its ligands (PD-L1 and PD-L2) on T cells (79), NK cells (79), neutrophils and monocytes (80) increase significantly in patients. Blocking the PD-1/PD-L signaling pathway significantly enhances the proliferative capacity of CD4^+^ T cells and the phagocytic and intracellular killing activity of macrophages, and reduces the Mtb content in the tissues of TB-affected mice (81). Rosemary V Swanson et al. found that antigen-specific B cells enhance cytokine production, strategically localize TFH-like cells within GrALT via PD-1-mediated interactions, and mediate Mtb control in both mice and macaques (28). However, some studies have shown that blocking the PD-1 signaling pathway can cause or exacerbate TB (82, 83), and that the TB incidence in cancer patients increase after immune checkpoint inhibitor (ICI) treatment (84). Blocking the Tim-3 signaling pathway via TIM3 monoclonal antibodies can increase the production of IL-2, IFN-γ, and TNF by T cells and inhibit the proliferation of Mtb in mice (85). In other studies, it was found that the Tim3-Gal9 interaction activates macrophage and increases bactericidal activity by inducing caspase-1-dependent IL-1β secretion (86). These conflicting results may be related to the expression levels of immune checkpoint molecules and effector cells (87). Therefore, it is essential to further explore the mechanisms causing immune dysfunction in TB patients. In a study by Piccaro et al., Mtb antigen 85B was identified as the major antigen causing T-cell exhaustion (88). Moreover, mitochondrial dysfunction contributes to T-cell functional exhaustion, thereby protecting recipient mice from *Mtb* infection (89). Headley et al. restored T-cell function through Mito-transfer, enhancing the ability of the host to fight TB infection (90). At the present stage, clinical trials investigating immune checkpoint therapy for tuberculosis have predominantly focused on the PD-1/PD-L1 signaling pathway (CTRI/2020/03/023815 and ChiCTR2500095521), with preliminary findings yet to be publicly disclosed.

The above studies indicate that antibodies may control TB through various pathways, and the ultimate mechanism of functional antibodies is most likely a comprehensive effect involving the induction of Mtb apoptosis in macrophages and the regulation of the TB immune microenvironment. However, most of the above studies on the bactericidal mechanisms of TB antibodies have been on polyclonal antibodies, whose bactericidal effects are a combined result of various antibodies, making the elucidation of their bactericidal mechanism more challenging. Moreover, emerging technologies have facilitated investigations into the relationship between Mtb-specific humoral immune profiles and TB progression (91); however, key antibodies remain elusive, and the mechanisms governing humoral immunity warrant further exploration (**Figure 3C**). Additionally, the development of mucosal immunity-targeting combination vaccines has demonstrated promising potential (92). The lack of mechanistic study on the involvement of immune checkpoint molecules in TB immunity is the main reason for the conflicting clinical research results. A systematic analysis of the changes in immune checkpoint molecules during the course of TB and research with a focus on the TB-specific immune checkpoint molecules (54) and subcellular organelles (90) will aid in the elucidation of the cause of immune dysfunction in TB patients. Moreover, the combined application of single-cell sequencing technology and antibody sequencing technology (13) will provide a platform for evaluating lymphocyte dysfunction and screening key antigen-specific monoclonal antibodies. On this basis, new methods will be used to rapidly prepare monoclonal antibodies (93, 94) and bioengineering technology will be employed to modify TB antibodies (95) to enhance the effects of ADCC, providing an important tool for TB antibody therapy.

For antibody-based therapy, the primary existing problems include the heterogeneity of Mtb antigens, which leads to poor cross-protection of monoclonal antibodies against different Mtb lineages. Polyclonal antibodies have complex components and unstable bactericidal effects, while the large-scale production and modification of high-efficiency neutralizing antibodies are still technically restricted. Notably, immune checkpoint antibody therapy has conflicting clinical outcomes: blocking PD-1/PD-L1 pathway may induce Mtb reactivation or aggravate local inflammation in some patients, due to the dual regulatory role of immune checkpoints in TB immune microenvironment. Moreover, antibody neutralization *in vivo* will reduce the therapeutic effect of exogenous antibodies, which is also a key bottleneck restricting clinical application.

## 5 Vaccines and TB

**Figure 4:**
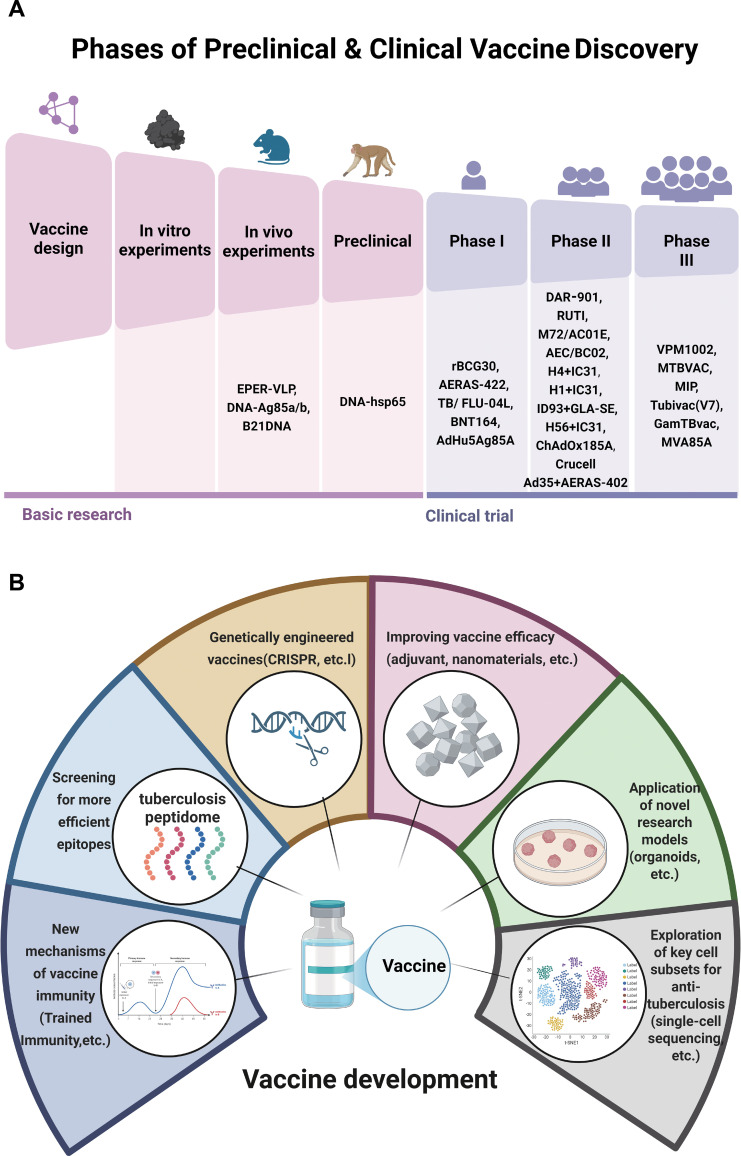
Current status of clinical trials and development directions of tuberculosis vaccines. **(A)** Current status of clinical trials for tuberculosis vaccines. **(B)** Development directions of tuberculosis vaccines.

Currently, more than 20 different types of TB vaccines have entered clinical research, including inactivated vaccines, live-attenuated vaccines (LAVs), protein/adjuvant vaccines, viral vector vaccines, and nucleic acid vaccines (**Figure 4A**, **Supplementary Table 3**) (96).

### 5.1 Live-attenuated vaccines

A goal of researchers is to develop LAVs with good safety and higher protective efficacy than the BCG vaccine (97, 98). Therefore, LAVs should lack virulence gene fragments but contain more immune antigen regions to induce more specific and longer-lasting immune responses (99). LAVs can be divided into recombinant BCG vaccines and gene-deleted attenuated Mtb strains.

#### 5.1.1 Recombinant BCG vaccines

Recombinant BCG vaccines have a stronger immunizing effect than the original BCG vaccine. These vaccines typically overexpress Mtb antigens missing in BCG (Ag85B (100, 101), Ag85A (101), CysVac (102) and RD1 region antigens), such as rBCG30 (phase I) (100) and AERAS-422 (101), or cytokines, such as VPM1002 (phase II) (103) and the auxotrophic recombinant BCG vaccine (104). rBCG30 was the first TB vaccine proven in preclinical studies to be more effective than the original BCG vaccine, but its development was terminated due to the presence of antibiotic resistance genes. VPM1002 is an attenuated strain that expresses listeriolysin (Hly) and lacks the urease C gene. It can stimulate strong Th17 and Th1 cell responses, induce more central memory T (Tcm) cells, enhance autophagy and inflammatory responses (105), and exhibit a stronger protective effect than the BCG vaccine (106).

#### 5.1.2 Gene-deleted attenuated Mtb strains

MtbVAC is an attenuated Mtb strain derived from the clinical isolate Mt103, with the deletion of two virulence genes, phoP and fadD26. It has become the only LAV to enter the human clinical trials (phase III) because of its good safety and protective efficacy (98, 107) The GC1237 mutant strain is a newly developed attenuated TB vaccine, which is a double-mutant strain of Rv1503c and PhoPR (108). This strain has safety similar to that of the BCG vaccine and provides good protection against the common Mtb lineage 4 (Euro-American) and lineage 2 (East Asia, Beijing) strains (108).

### 5.2 Inactivated vaccines

Inactivated TB vaccines mainly include MIP (phase III), DAR-901 (phase IIb), Tubivac (phase III), and RUTI (phase IIb). Due to the good immunogenicity and mature production process of inactivated TB vaccines, RUTI and DAR-901 are being tested in phase IIb clinical trials, and MIP and Tubivac are being tested in phase III clinical trials.

### 5.3 Recombinant protein subunit/adjuvant vaccines

The antigens currently used in protein subunit vaccine research mainly include early secreted proteins (e.g., ESAT-6, Ag85B, Ag85A, and MPT64) (109), cell wall proteins (e.g., heat shock protein HspX (110)), and cell wall-associated PE/PPE family proteins (111). The relevant adjuvants mainly include IC31 (112), GLA-SE (113), AS01E (110), CpG (114) and BC02 (115). At present, eight recombinant protein subunit/adjuvant vaccines have entered clinical trials, and multiple vaccines have shown good safety and efficacy in clinical trials (110, 113, 116).

### 5.4 Nucleic acid vaccines

TB nucleic acid vaccines are mainly divided into DNA vaccines and RNA vaccines. The DNA vaccines currently under study include DNA-hsp65 (117), DNA-Ag85a/b (118) and B21 (119) DNA vaccines, which have all exhibited cellular immune responses and induced enhanced effector memory T-cell and Tcm-cell generation in these studies. A TB mRNA prophylactic vaccine (BNT164a1/BNT164b1, BioNTech) recently entered phase I clinical trials in Germany (NCT05547464) and South Africa (NCT05547464) (120).

### 5.5 Viral vector vaccines

At present, the TB antigens loaded in viral vector vaccines include Ag85A and ESAT6, and the viruses used include adenovirus, influenza virus, cytomegalovirus, Sendai virus, and vesicular stomatitis virus (121). There are eight main vaccines that have entered clinical trials, including ChAdOx1 85A (phase II) (122), MVA85A(phase II b) (121, 122), TB/FLU-04L ((phase I) (121) and Crucell Ad35^+^AERAS-402(phase II) (121). In addition, vaccines such as Ad5Ag85A (phase II) and MVA85A (phase IIb) have demonstrated the ability to induce strong Th1-type and Th17-type CD4^+^ T-cell immune responses in clinical trials (121). Moreover, a variety of other vaccines have shown good efficacy in animal models (121), greatly increasing the success rate of TB viral vector vaccines.

Current TB vaccine candidates also have obvious limitations and potential problems. Traditional BCG has unstable protective effect in adults and cannot prevent latent TB reactivation. Most novel vaccines based on classic antigens (Ag85, ESAT-6) fail to achieve ideal protective efficacy due to Mtb antigen variation. In clinical trials, individual vaccines may cause local redness, swelling and low-grade fever after inoculation. In addition, the gut microbiota status of recipients will interfere with vaccine immune response, and there is still a lack of unified evaluation criteria for vaccine protective efficacy and mature animal models for efficacy verification.

So far, significant progress has been made in the research on new TB vaccines. However, challenges remain in areas such as antigen selection, protective efficacy evaluation indicators, and animal models. Moreover, the concept of trained immunity (123) has expanded the target cells for vaccine action. At present, vaccine development still mainly focuses on classic antigens such as Ag85 and ESAT. With the continuous development of bioinformatic and immunological techniques, antigen epitope prediction technology has become increasingly developed, and the screening of new and more efficient antigenic epitopes is key to breakthroughs in vaccine development. Enhancing the efficacy of existing vaccines constitutes another critical research avenue, as Bidyarani et al. proposed that the application of nanotechnology or DNA-based technologies could improve tuberculosis vaccine performance (124). On the one hand, experimental studies have shown that the immunogenicity of certain epitope regions of Mtb is better than that of the whole antigen (125). The efficacy of existing antigen vaccines can by enhanced by using CRISPR (126) genetic engineering technology to recombine and construct virus-like particle (VLP) vaccines and modify antigens (127). Additionally, protein vaccines are often combined with immune adjuvants, but the limited variety of adjuvants currently approved for human vaccines restricts the ability to increase the immunogenicity of existing vaccines by the addition of adjuvants. Additionally, research has revealed that the gut affects vaccine efficacy (128). Therefore, developing adjuvants for TB vaccines or using nanomaterials to load antigens can further increase vaccine efficacy (129, 130). Studies around BCG have also found that changing the vaccination method can improve the protective efficacy of the vaccine, intravenous infusion of BCG has been shown to enhance the protective efficacy in mouse models, and BCG inoculation by nebulization has also been found to have a good safety profile in clinical trials and early bacterial clearance (NCT04777721) (131). In addition, novel research models, such as controlled human infection models and ultralow-dose aerosol infection mouse models, will also be help screen more reliable TB vaccines in the preclinical stage (121) (**Figure 4B**).

## 6 Cytokine therapy and TB

**Figure 5:**
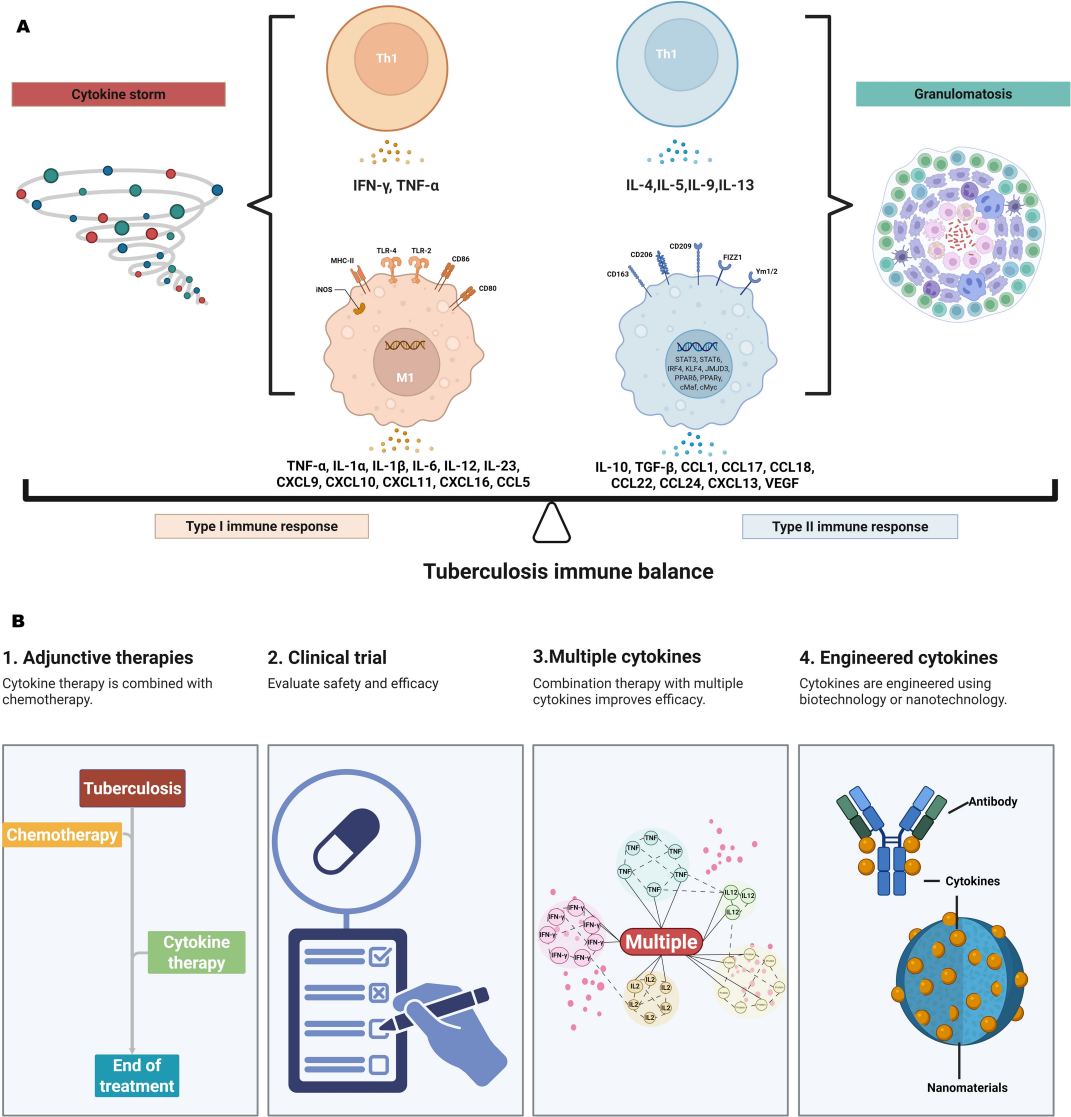
Mechanisms and development directions of cytokine therapy for tuberculosis. **(A)** Cytokine-mediated immune balance in tuberculosis. **(B)** Development directions of cytokine therapy for tuberculosis.

In TB, it is very important to prevent the protective inflammatory immune responses from becoming a chronic pathological inflammatory response, and this prevention largely depends on the expression of cytokines (132). Type I immune response cytokines (IL-1, TNF-α (133), IFN-γ (134) and IL-17A) are the main effector cytokines that mediate protective immunity but can also cause tissue damage and pathological reactions. Anti-inflammatory cytokines such as IL-4, IL-10 and TGF-β control immune damage by reducing inflammation, and IL-27-mediated immunosuppression can significantly alleviate structural damage in lung granulomas (7) (**Figure 5A**). Therefore, cytokines are a double-edged sword. The rational use of cytokines in Mtb infection is crucial for TB treatment.

Cytokine therapies for TB treatment have already entered clinical trials(**Supplementary Table 4**). IFN-γ therapy as an adjuvant helps improve respiratory symptoms, increase sputum negative conversion rates, and reduces adverse reactions in patients with pulmonary TB or MDR-TB (135). IFNγ-secreting T cells that highly express IL-2 potently inhibit the growth of intracellular Mtb in macrophages (136), and the use of IL-2 in TB treatment has demonstrated that adjuvant IL-2 therapy for MDR-TB is highly safe, increases sputum smear conversion rates, and alleviates lung lesions (3). Studies have demonstrated that GM-CSF mutations are associated with increased susceptibility to tuberculosis, whereas in animal experiments, recombinant GM-CSF adenovirus can induce DC activation, enhance IFN-γ secretion, and reduce pulmonary bacterial load in mice (133, 137). And in the IL-6 study, researchers investigated altered IL-6 signalling and the risk of TB (138). Cytokine combination therapy has also been developed for TB, and studies have shown that using IFN-γ and IgA antibodies in Mtb-infected mice reduces lung pathology, decreases lung granulomatous infiltration, and lowers the Mtb load (75). The same studies also found that TNF-α antagonist therapy is associated with an increased risk of TB disease (139), whereas IL-17 or IL-2 enhances immune control of Mtb infection and is linked to increased nitric oxide (NO) production (16). These findings indicate that some cytokines can be used as adjuvant therapy for MDR-TB and that the prophylactic use of therapeutic cytokines can help prevent post-treatment relapse caused by drug-resistant Mtb.

The proinflammatory and anti-inflammatory effects of cytokines determine their dual roles in TB. Moreover, the course and progression of TB affect the types of cytokine treatment methods that should be implemented. At present, most clinical trials of cytokine therapy for TB are in the preclinical, phase I/II stages. Only the trials NCT03069534 and NCT04766307 using IL-2 (140) have progressed to phases III and IV, but the results have not yet been published. Existing studies have found that a variety of cytokines play roles in fighting TB infection; therefore, treatment with a single cytokine may not achieve the desired effect. The relationships among cytokines in the context of TB should be further studied, systematic clinical trials of cytokine combinations or cytokines combined with chemotherapy should be carried out, and the targeting properties of monoclonal antibodies in cancer treatment should be leveraged to specifically guide cytokines to the intended site to yield better outcomes while avoiding the systemic toxicity of free cytokines (141). Similar to cancer treatment, attention should be given to the side effects of cytokine therapy, with the most severe being “cytokine storm,” which can lead to systemic damage, multiorgan failure, and death. Besides cytokine storm, unregulated application of single pro-inflammatory cytokines will break the balance of Th1/Th2 immune response, aggravate lung granuloma and tissue damage. Most cytokines have short half-life *in vivo* and lack tissue targeting, resulting in low local concentration in lung lesions and obvious systemic side effects. At present, the dosage, administration cycle and combination scheme of cytokine adjuvant therapy have not formed unified clinical specifications, and the long-term safety of repeated medication remains to be verified.Therefore, cytokine therapy presents both opportunities and challenges for the clinical treatment of TB(**Figure 5B**). The rational and standardized use of cytokines is critical for TB treatment.

## 7 Microbial therapy and TB

**Figure 6:**
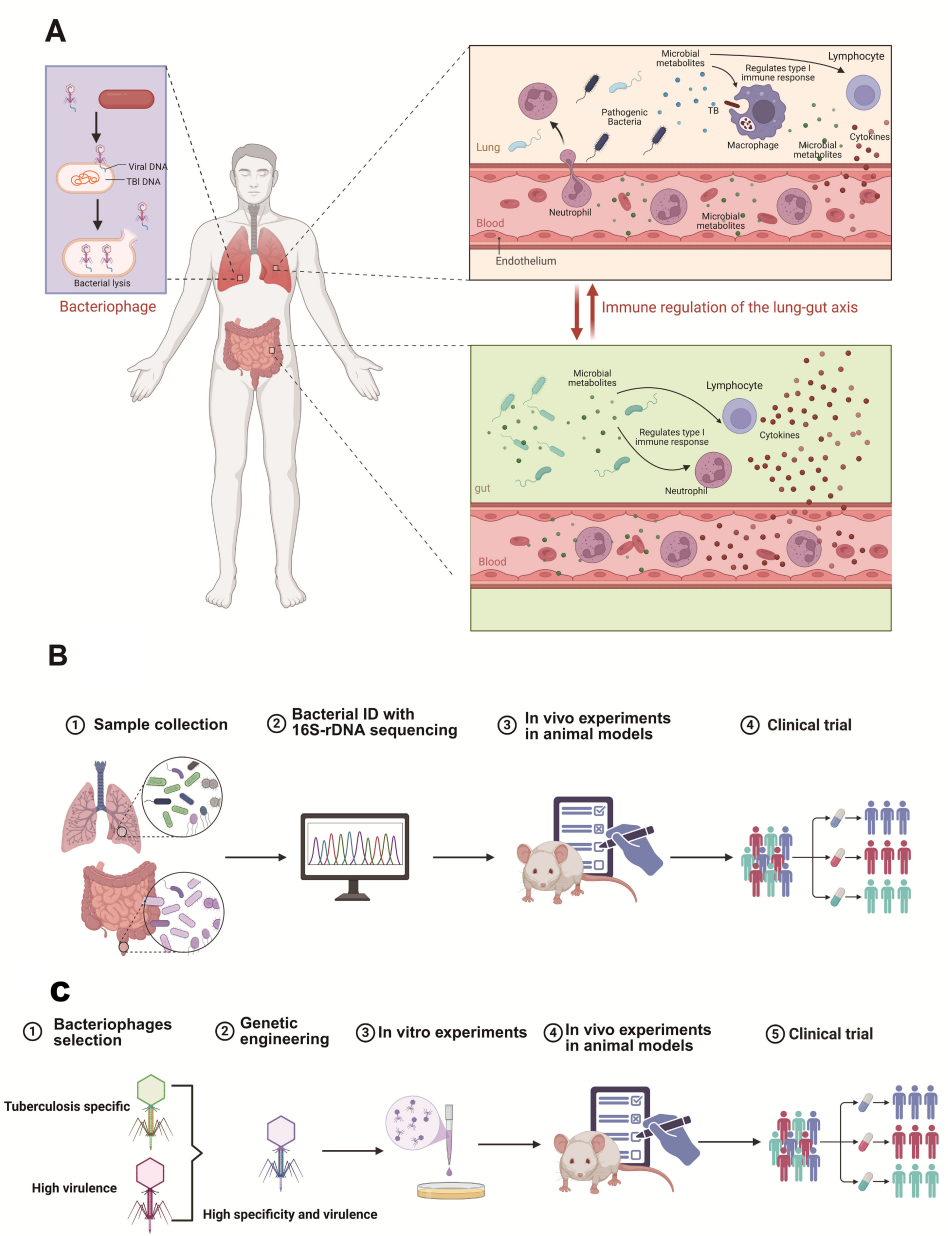
Mechanisms and development directions of microbial therapy for tuberculosis. **(A)** Mechanisms of microbial therapy for tuberculosis. **(B)** Development directions of microbiota therapy for tuberculosis. **(C)** Development directions of phage therapy for tuberculosis.

The gut microbiota and the host have a complex relationship. On the one hand, disrupted interactions between the gut microbiota and immune system leads to flora dysbiosis, impaired immunity (142), promotes Mycobacteria pulmonary colonization (143), increasing the risk of TB. By producing metabolites (142) and regulating the host immune system (144, 145), gut microbes can also directly or indirectly inhibit the growth of or kill Mtb (146, 147) (**Figure 6A**). Mtb infection can alter the bacterial abundance and α- and β-diversity of the host’s gut and lung microbiota (148, 149), thereby affecting the host’s susceptibility to TB (150). The diversity of gut microbiota, the abundance of intermediate metabolites, and microbial interactions in drug-resistant TB patients change according to the type of antibiotics used during treatment, thus affecting the treatment outcomes of patients (151). Chai et al. found that the gut microbiota affects the infection progression and treatment outcomes in TB patients by regulating the proportion of CD4^+^/CD8^+^ T cells and the levels of the inflammatory cytokines IL-2, IL-4 and IL-10 in the intestine (152). Yang et al. reported that oral administration of *Bacteroides fragilis* increases the expression of lncRNA-CGB and promotes anti-TB immunity (146). Kaufmann et al. identified that indolepropionate, one of the metabolic products of gut bacteria—*Clostridium sporogenes*, can inhibit Mtb proliferation by enhancing the phagocytic activity of macrophages, and the results showed that this treatment was more effective than anti-TB drugs (153). Probiotic PMC205 induced recovery of disrupted lung microflora, increased butyric acid, suppressed excessive inflammation to improve survival and promoted autophagy (154). Moreover, supplementation with *Lactobacillus casei* during the intensive treatment stage of TB can significantly reduce the concentrations of inflammatory cytokines TNF-α, IL-6, IL-10, and IL-12 and increase the concentrations of plasma metabolites such as phosphatidylserine, horsesin 1, phosphatidylcholine, L-glycine and pyridoxamine to improve the clinical symptoms and treatment outcomes of TB patients (155). Burrows demonstrated that intestinal transplantation of *Tritrichomonas* spp. promotes the lung infiltration of ILC2s cells and reduces the Mtb load in the lungs (156)(**sTable 5**). It has also been found that the BCG vaccine can alter the intestinal microbiota, which induces lung-resident memory macrophages and trained immunity via the gut–lung axis (123).

Phage therapy is a supplementary regimen for the treatment of drug-resistant bacteria and is feasible for the treatment of MDR-TB (157, 158), and potential phages have been screened (159)(**Figure 6A**). Researchers have selected effective phage mixtures through *in vitro* experiments (160) and further demonstrated the effective Mtb-killing ability of phage DS6A (161), D29 LysB (162), Henu3 (163) and KSSH1 (164), thus confirming the potential value of phages in TB treatment. The lytic phage KVT1 modified by Nayak et al. using bioengineering methods exhibits high specificity and is a strong candidate for phage therapy for TB (165). Wang et al. engineered a *Mtb* strain with a tripartite killing mechanism to enable controlled tuberculosis management (166). In addition, Dedrick et al. used phage therapy in 20 patients with drug-resistant mycobacterial infections, and their results showed that that no adverse reactions were observed in any patient despite variations in pathogens, phage species, and administration routes; however, four patients had poor therapeutic outcomes, which might be attributed to antibody-mediated neutralization reactions in patients (167) (**Supplementary Table 5**).

Microbial therapy demonstrates high specificity and safety, effectively avoiding the side effects and drug resistance problems caused by antibiotics. Both the regulation of gut microbiota and the use of phages for treatment have broad application prospects in TB treatment. At present, the changes in the gut microbiota of TB patients during different stages of the disease have been characterized (149). Further systematic analysis of the relationship between the microbiota and TB in the lung microenvironment and the development of clinical application research will promote the application of microbes in TB treatment. Using sequencing and other methods to detect changes in microbiota composition in the lung tissue and the gut during the TB process, analyze the microbiota interactions between the lungs and the gut, and explore the impact of key microbial metabolites on immune function will accumulate experimental data that support the use of probiotics in TB treatment, prevention, and rehabilitation. The efficacy of phage therapy can be further improved by using bioengineering techniques to screen and modify phages to further enhance their specificity and lytic ability and reduce their immunogenicity. It is also highly necessary to simultaneously conduct systematic animal experiments, clinical application research on the combination of existing clinical probiotics with chemical drugs, and administration method (168), as well as evaluate the safety and efficacy of microbial therapy (**Figure 6B**). Through in-depth research and development, microbial therapy is expected to become an effective, safe and economical treatment method for TB, providing new ideas and approaches for the control and treatment of TB.

At the same time, microbial therapy including probiotics and phage therapy also has practical challenges. The composition of gut microbiota varies greatly among individuals, so the intervention effect of probiotics is individualized and unstable. Oral probiotics are easily affected by gastric acid and intestinal environment, resulting in low effective colonization rate. For phage therapy, phages are easy to induce host antibody neutralization, and Mtb can generate phage-resistant strains after long-term use. In addition, phage preparations have poor thermal stability, and the aerosol administration method for pulmonary TB still needs to be optimized for safety and operability.

## 8 Future development of immunotherapy for TB

The emergence and prevalence of Multidrug-resistant tuberculosis (MDR-TB) have significantly undermined the efficacy of conventional treatments, while the success experience of cancer immunotherapy and advancements in biotechnologies have collectively driven the rapid development of TB immunotherapy. Single-cell sequencing has revealed transcriptomic heterogeneity of immune cells (60) and associations between lymphocyte development and intercellular networks during TB infection (169), PhIP-seq facilitates the screening of novel neutralizing antibodies (170), the IsoPlexis provides a platform for the discovery of “superimmune cells”, and the identified roles of mitophagy (171) and fatty acid metabolism (172) in TB progression suggest that TB pathogenesis results from the combined action of multiple immune regulatory mechanisms. The proposal of novel immunological theories in recent years, along with the discovery of new anti-infection mechanisms such as proteasome-derived defence peptides (173), has additionally offered new perspectives for the immunotherapy of tuberculosis.

However, current TB immunotherapy faces multiple challenges: existing vaccines focusing on classical antigens (Ag85 and ESAT6) have failed to achieve desired protective efficacy, contradictory findings in immune checkpoint research, limited application of cell therapies (insufficient key antigen research and safety concerns), stringent experimental animal conditions and scarce systematic studies. To address these bottlenecks, future research should focus on: using single-cell sequencing and antibody-omics to analyze key TCR and BCR clonotypes involved in TB occurrence, systematically investigating lung microenvironment checkpoints driving immune exhaustion and validating therapeutic targets, exploring low-toxicity approaches like γδT-cell and CIK cell therapies, developing safe CAR-T strategies leveraging Mtb antigen conservation, enhancing chemotherapy efficacy through immunomodulator-antibiotic combinations and reducing recurrence via immunomodulator-probiotic synergy using nutritional immunity (156).

Existing reviews have predominantly centered on vaccine development (174), phage therapy, and TB immune mechanisms, providing recommendations for optimizing delivery systems, efficacy, and safety profiles (175). In contrast, this review systematically integrates immune cell-based therapies, antibody-mediated interventions, and probiotic-based approaches for the first time in the field, addressing a critical gap in current literature. It highlights the utility of cutting-edge technologies-including single-cell sequencing, PhIP-seq, and IsoPlexis platforms-in dissecting immune heterogeneity (e.g., TCR/BCR clonotypes) and screening for “superimmune cells.” Emerging technologies such as high-parameter flow cytometry and spatial transcriptomics hold promise for identifying novel immunotherapeutic targets and formulating innovative strategies, while systematic clinical trials of multitargeted and antibiotic-combination therapies could be facilitated using organoid models (176) and animal studies (177). Additionally, the review proposes that leveraging advancements in cancer immunotherapy may offer instructive parallels for TB immunotherapy. These endeavors may not only yield new approaches for TB immunotherapy but also expand therapeutic arsenals for future MDR-TB management.

In conclusion, breakthroughs in TB immunotherapy hinge upon the integration of fundamental mechanistic research and technological innovation; through the integration of multi-omics data, precise targeting of immune checkpoints, optimization of cell therapy safety profiles, and advancement of clinical trials for combination strategies, a paradigm shift from “anti-infection” to “immune modulation” in TB treatment could potentially be realized.

## References

[1] Sonali SP-S Sanket SR Sohan SC . Reimagining tuberculosis treatment: The promise of immunotherapy and drug repurposing. Curr Pharmacogenomics Pers Med. (2025) 22:231–50. doi: 10.2174/0118756921381395250614152438

[2] Fatima S Kumari A Das G Dwivedi VP . Tuberculosis vaccine: A journey from BCG to present. Life Sci. (2020) 252:117594. doi: 10.1016/j.lfs.2020.117594 32305522

[3] Nie W Wang J Zeng J Wang Q Du Y Tan Q . Adjunctive interleukin-2 for the treatment of drug-susceptible tuberculosis: A randomized control trial in China. Infection. (2022) 50:413–21. doi: 10.1007/s15010-021-01698-3 34562262

[4] Goletti D Meintjes G Andrade BB Zumla A Shan Lee S . Insights from the 2024 WHO Global Tuberculosis Report - more comprehensive action, innovation, and investments required for achieving WHO End TB goals. Int J Infect Dis. (2024) 150:107325. doi: 10.1016/j.ijid.2024.107325 39631498

[5] Trajman A Campbell JR Kunor T Ruslami R Amanullah F Behr MA . Tuberculosis. Lancet. (2025) 405:850–66. doi: 10.1016/s0140-6736(24)02479-6 40057344

[6] Lyu MY Lai HL Peng HR Luo H Zhou J Ma WA . Immunotherapy for tuberculosis: Current strategies and future directions. Mil Med Res. (2025) 12:68. doi: 10.1186/s40779-025-00655-7 41116166 PMC12536540

[7] Scriba TJ Maseeme M Young C Taylor L Leslie AJ . Immunopathology in human tuberculosis. Sci Immunol. (2024) 9:eado5951. doi: 10.1126/sciimmunol.ado5951 39671470

[8] Krishnan V Nath S Nair P Das B . Mycobacterium tuberculosis and its clever approaches to escape the deadly macrophage. World J Microbiol Biotechnol. (2023) 39:300. doi: 10.1007/s11274-023-03735-9 37667129

[9] Ghanavi J Farnia P Farnia P Velayati AA . The role of interferon-gamma and interferon-gamma receptor in tuberculosis and nontuberculous mycobacterial infections. Int J Mycobacteriol. (2021) 10:349–57. doi: 10.4103/ijmy.ijmy_186_21 34916451

[10] Feng S McNehlan ME Kinsella RL Sur Chowdhury C Chavez SM Naik SK . Autophagy promotes efficient T cell responses to restrict high-dose Mycobacterium tuberculosis infection in mice. Nat Microbiol. (2024) 9:684–97. doi: 10.1038/s41564-024-01608-x 38413834 PMC12665381

[11] Laopanupong T Prombutara P Kanjanasirirat P Benjaskulluecha S Boonmee A Palaga T . Lysosome repositioning as an autophagy escape mechanism by Mycobacterium tuberculosis Beijing strain. Sci Rep. (2021) 11:4342. doi: 10.1038/s41598-021-83835-4 33619301 PMC7900199

[12] Verma A Ghoshal A Dwivedi VP Bhaskar A . Tuberculosis: The success tale of less explored dormant Mycobacterium tuberculosis. Front Cell Infect Microbiol. (2022) 12:1079569. doi: 10.3389/fcimb.2022.1079569 36619761 PMC9813417

[13] Lyu M Xu G Zhou J Reboud J Wang Y Lai H . Single-cell sequencing reveals functional alterations in tuberculosis. Adv Sci (Weinh). (2024) 11:e2305592. doi: 10.1002/advs.202305592 38192178 PMC10953544

[14] Cohen SB Gern BH Urdahl KB . The tuberculous granuloma and preexisting immunity. Annu Rev Immunol. (2022) 40:589–614. doi: 10.1146/annurev-immunol-093019-125148 35130029

[15] Bromley JD Ganchua SKC Nyquist SK Maiello P Chao M Borish HJ . CD4(+) T cells re-wire granuloma cellularity and regulatory networks to promote immunomodulation following Mtb reinfection. Immunity. (2024) 57:2380–98:e2386. doi: 10.1016/j.immuni.2024.08.002 39214090 PMC11466276

[16] Ogongo P Tezera LB Ardain A Nhamoyebonde S Ramsuran D Singh A . Tissue-resident-like CD4+ T cells secreting IL-17 control Mycobacterium tuberculosis in the human lung. J Clin Invest. (2021) 131(10):e142014. doi: 10.1172/JCI142014 33848273 PMC8121523

[17] Abdul-Aziz AA Elhassan MM Yousuf AM Hamid ME Abdulsalam SA Gafar RA . Changes of th1 and th2 cytokines levels among Sudanese tuberculosis patients during treatment. Int J Mycobacteriol. (2022) 11:70–4. doi: 10.4103/ijmy.ijmy_245_21 35295026

[18] Vats D Rani G Arora A Sharma V Rathore I Mubeen SA . Tuberculosis and T cells: Impact of T cell diversity in tuberculosis infection. Tuberculosis (Edinb). (2024) 149:102567. doi: 10.1016/j.tube.2024.102567 39305817

[19] Zumwinkel M Chirambo A Zahnle M Burger M Grieshober M Romahn V . Polycytotoxic T cells mediate antimicrobial activity against intracellular Mycobacterium tuberculosis. Infect Immun. (2024) 93(1):e0029724. doi: 10.1128/iai.00297-24 39660897 PMC11784352

[20] Lu YJ Barreira-Silva P Boyce S Powers J Cavallo K Behar SM . CD4 T cell help prevents CD8 T cell exhaustion and promotes control of Mycobacterium tuberculosis infection. Cell Rep. (2021) 36:109696. doi: 10.1016/j.celrep.2021.109696 34525366 PMC8466141

[21] Scriba TJ Netea MG Ginsberg AM . Key recent advances in TB vaccine development and understanding of protective immune responses against Mycobacterium tuberculosis. Semin Immunol. (2020) 50:101431. doi: 10.1016/j.smim.2020.101431 33279383 PMC7786643

[22] Andrews JT Zhang Z Prasad G Huey F Nazarova EV Wang J . Metabolically active neutrophils represent a permissive niche for Mycobacterium tuberculosis. Mucosal Immunol. (2024) 17:825–42. doi: 10.1016/j.mucimm.2024.05.007 38844208 PMC11493682

[23] Sankar P Ramos RB Corro J Mishra LK Nafiz TN Bhargavi G . Fatty acid metabolism in neutrophils promotes lung damage and bacterial replication during tuberculosis. PloS Pathog. (2024) 20:e1012188. doi: 10.1371/journal.ppat.1012188 39365825 PMC11482725

[24] Ahor HS Vivekanandan MM Adankwah E Minadzi D Acheampong I Aniagyei W . Monocyte transcriptome signatures of inflammation and enhanced neutrophil recruitment characterize immunopathology in the blood of tuberculosis patients. J Infect. (2024) 89:106359. doi: 10.1016/j.jinf.2024.106359 39603348

[25] Balboa L Barrios-Payan J Gonzalez-Dominguez E Lastrucci C Lugo-Villarino G Mata-Espinoza D . Diverging biological roles among human monocyte subsets in the context of tuberculosis infection. Clin Sci (Lond). (2015) 129:319–30. doi: 10.1042/CS20150021 25858460

[26] Abebe F . Immunological basis of early clearance of Mycobacterium tuberculosis infection: The role of natural killer cells. Clin Exp Immunol. (2021) 204:32–40. doi: 10.1111/cei.13565 33315236 PMC7944356

[27] Wang Q Nag D Baldwin SL Coler RN McNamara RP . Antibodies as key mediators of protection against Mycobacterium tuberculosis. Front Immunol. (2024) 15:1430955. doi: 10.3389/fimmu.2024.1430955 39286260 PMC11402706

[28] Swanson RV Gupta A Foreman TW Lu L Choreno-Parra JA Mbandi SK . Antigen-specific B cells direct T follicular-like helper cells into lymphoid follicles to mediate Mycobacterium tuberculosis control. Nat Immunol. (2023) 24:855–68. doi: 10.1038/s41590-023-01476-3 37012543 PMC11133959

[29] Carpenter SM Lu LL . Leveraging antibody, B cell and Fc receptor interactions to understand heterogeneous immune responses in tuberculosis. Front Immunol. (2022) 13:830482. doi: 10.3389/fimmu.2022.830482 35371092 PMC8968866

[30] Kim SJ Karamooz E . MR1- and HLA-E-dependent antigen presentation of Mycobacterium tuberculosis. Int J Mol Sci. (2022) 23(22):14412. doi: 10.3390/ijms232214412 36430890 PMC9693577

[31] Wei L Liu L Meng Z Qi K Gao X Feng J . Recognition of Mycobacterium tuberculosis by macrophage Toll-like receptor and its role in autophagy. Inflammation Res. (2024) 73:753–70. doi: 10.1007/s00011-024-01864-x 38563966

[32] Vorkas CK Levy O Skular M Li K Aube J Glickman MS . Efficient 5-OP-RU-induced enrichment of mucosa-associated invariant T cells in the murine lung does not enhance control of aerosol Mycobacterium tuberculosis infection. Infect Immun. (2020) 89(1):e00524–20. doi: 10.1128/IAI.00524-20 33077620 PMC7927919

[33] Xiong K Sun W Wang H Xie J Su B Fan L . The frequency and dynamics of CD4(+) mucosal-associated invariant T (MAIT) cells in active pulmonary tuberculosis. Cell Immunol. (2021) 365:104381. doi: 10.1016/j.cellimm.2021.104381 34049011

[34] Zhou CY Yang YL Han ZY Chen YX Liu HL Fan K . Peripheral blood MR1 tetramer-positive mucosal-associated invariant T-cell function is modulated by mammalian target of rapamycin complex 1 in patients with active tuberculosis. Immunology. (2024) 173:497–510. doi: 10.1111/imm.13834 39022997

[35] Rampoldi F Ullrich L Prinz I . Revisiting the interaction of γδ T-cells and B-cells. Cells. (2020) 9(3):743. doi: 10.3390/cells9030743 32197382 PMC7140609

[36] Wei J Guo F Song Y Feng T Wang Y Xu K . Analysis of the components of Mycobacterium tuberculosis heat-resistant antigen (Mtb-HAg) and its regulation of γδ T-cell function. Cell Mol Biol Lett. (2024) 29:70. doi: 10.1186/s11658-024-00585-7 38741147 PMC11089708

[37] Corral D Charton A Krauss MZ Blanquart E Levillain F Lefrancais E . ILC precursors differentiate into metabolically distinct ILC1-like cells during Mycobacterium tuberculosis infection. Cell Rep. (2022) 39:110715. doi: 10.1016/j.celrep.2022.110715 35443177 PMC9043616

[38] Ardain A Domingo-Gonzalez R Das S Kazer SW Howard NC Singh A . Group 3 innate lymphoid cells mediate early protective immunity against tuberculosis. Nature. (2019) 570:528–32. doi: 10.1038/s41586-019-1276-2 31168092 PMC6626542

[39] Das S Chauhan KS Ahmed M Akter S Lu L Colonna M . Lung type 3 innate lymphoid cells respond early following Mycobacterium tuberculosis infection. mBio. (2024) 15:e0329923. doi: 10.1128/mbio.03299-23 38407132 PMC11005430

[40] Cronan MR Hughes EJ Brewer WJ Viswanathan G Hunt EG Singh B . A non-canonical type 2 immune response coordinates tuberculous granuloma formation and epithelialization. Cell. (2021) 184:1757–74:e1714. doi: 10.1016/j.cell.2021.02.046 33761328 PMC8055144

[41] Nouari W Aribi M . Innate lymphoid cells, immune functional dynamics, epithelial parallels, and therapeutic frontiers in infections. Int Rev Immunol. (2025) 44(5):245–72. doi: 10.1080/08830185.2025.2490233 40242974

[42] Grassi G Vanini V De Santis F Romagnoli A Aiello A Casetti R . PMN-MDSC frequency discriminates active versus latent tuberculosis and could play a role in counteracting the immune-mediated lung damage in active disease. Front Immunol. (2021) 12:594376. doi: 10.3389/fimmu.2021.594376 33981297 PMC8107479

[43] Darrah PA Zeppa JJ Maiello P Hackney JA Wadsworth MH, 2nd Hughes TK . Prevention of tuberculosis in macaques after intravenous BCG immunization. Nature. (2020) 577:95–102. doi: 10.1038/s41586-019-1817-8 31894150 PMC7015856

[44] Nanda N Alphonse MP . From host defense to metabolic signatures: Unveiling the role of γδ T cells in bacterial infections. Biomolecules. (2024) 14(2):225. doi: 10.3390/biom14020225 38397462 PMC10886488

[45] Liang J Fu L Li M Chen Y Wang Y Lin Y . Allogeneic Vγ9Vδ2 T-cell therapy promotes pulmonary lesion repair: An open-label, single-arm pilot study in patients with multidrug-resistant tuberculosis. Front Immunol. (2021) 12:756495. doi: 10.3389/fimmu.2021.756495 34975844 PMC8715986

[46] Kim H Shin SJ . Pathological and protective roles of dendritic cells in Mycobacterium tuberculosis infection: Interaction between host immune responses and pathogen evasion. Front Cell Infect Microbiol. (2022) 12:891878. doi: 10.3389/fcimb.2022.891878 35967869 PMC9366614

[47] Tang P Chen X Xu J Hu Y Ye Z Wang X . Autologous cytokine-induced killer cell immunotherapy enhances chemotherapy efficacy against multidrug-resistant tuberculosis. J Immunol Res. (2022) 2022:2943113. doi: 10.1155/2022/2943113 35340584 PMC8947923

[48] Paterson RL La Manna MP Arena De Souza V Walker A Gibbs-Howe D Kulkarni R . An HLA-E-targeted TCR bispecific molecule redirects T cell immunity against Mycobacterium tuberculosis. Proc Natl Acad Sci USA. (2024) 121:e2318003121. doi: 10.1073/pnas.2318003121 38691588 PMC11087797

[49] Sterner RC Sterner RM . CAR-T cell therapy: current limitations and potential strategies. Blood Cancer J. (2021) 11:69. doi: 10.1038/s41408-021-00459-7 33824268 PMC8024391

[50] Liu E Marin D Banerjee P Macapinlac HA Thompson P Basar R . Use of CAR-transduced natural killer cells in CD19-positive lymphoid tumors. N Engl J Med. (2020) 382:545–53. doi: 10.1056/NEJMoa1910607 32023374 PMC7101242

[51] Klichinsky M Ruella M Shestova O Lu XM Best A Zeeman M . Human chimeric antigen receptor macrophages for cancer immunotherapy. Nat Biotechnol. (2020) 38:947–53. doi: 10.1038/s41587-020-0462-y 32361713 PMC7883632

[52] Voogd L Drittij A Dingenouts CKE Franken K Unen VV van Meijgaarden KE . Mtb HLA-E-tetramer-sorted CD8(+) T cells have a diverse TCR repertoire. iScience. (2024) 27:109233. doi: 10.1016/j.isci.2024.109233 38439958 PMC10909886

[53] Shekarkar Azgomi M Badami GD Lo Pizzo M Tamburini B Dieli C La Manna MP . Integrated analysis of single-cell and bulk RNA sequencing data reveals memory-like NK cell subset associated with Mycobacterium tuberculosis latency. Cells. (2024) 13(4):293. doi: 10.3390/cells13040293 38391906 PMC10886487

[54] Wang J Chai Q Lei Z Wang Y He J Ge P . LILRB1-HLA-G axis defines a checkpoint driving natural killer cell exhaustion in tuberculosis. EMBO Mol Med. (2024) 16:1755–90. doi: 10.1038/s44321-024-00106-1 39030302 PMC11319715

[55] Dai Y Wang X Du W Chen R Ma F Ma T . NK cell-derived exosomes inhibit survival of Mycobacterium tuberculosis by promoting apoptosis in mice. Cytokine. (2025) 185:156820. doi: 10.1016/j.cyto.2024.156820 39612656

[56] Narinyan W Poladian N Orujyan D Gargaloyan A Venketaraman V . Immunologic role of innate lymphoid cells against Mycobacterial tuberculosis infection. Biomedicines. (2022) 10(11):2828. doi: 10.3390/biomedicines10112828 36359348 PMC9687238

[57] Kathamuthu GR Pavan Kumar N Moideen K Dolla C Kumaran P Babu S . Multi-dimensionality immunophenotyping analyses of MAIT cells expressing Th1/Th17 cytokines and cytotoxic markers in latent tuberculosis diabetes comorbidity. Pathogens. (2022) 11(1):87. doi: 10.3390/pathogens11010087 35056035 PMC8777702

[58] Dey RJ Dey B Harriff M Canfield ET Lewinsohn DM Bishai WR . Augmentation of the riboflavin-biosynthetic pathway enhances mucosa-associated invariant T (MAIT) cell activation and diminishes Mycobacterium tuberculosis virulence. mBio. (2021) 13:e0386521. doi: 10.1128/mbio.03865-21 35164552 PMC8844931

[59] La Manna MP Orlando V Tamburini B Badami GD Dieli F Caccamo N . Harnessing unconventional T cells for immunotherapy of tuberculosis. Front Immunol. (2020) 11:2107. doi: 10.3389/fimmu.2020.02107 33013888 PMC7497315

[60] Zhan Y Zhang Q Wang W Liang W Wang C . Single-cell RNA sequencing in tuberculosis: application and future perspectives. Chin Med J (Engl). (2024) 138(14):1676–86. doi: 10.1097/cm9.0000000000003095 39111829 PMC12273648

[61] Li H Ji J Qu M Ma X Zuo Y Tang M . Isolation and characterization of a protective monoclonal antibody targeting outer membrane protein (OmpA) against tuberculosis. Microbiol Spectr. (2025) 13:e0294224. doi: 10.1128/spectrum.02942-24 39964152 PMC11960079

[62] Abebe F Bjune G . The protective role of antibody responses during Mycobacterium tuberculosis infection. Clin Exp Immunol. (2009) 157:235–43. doi: 10.1111/j.1365-2249.2009.03967.x 19604263 PMC2730849

[63] Guirado E Amat I Gil O Diaz J Arcos V Caceres N . Passive serum therapy with polyclonal antibodies against Mycobacterium tuberculosis protects against post-chemotherapy relapse of tuberculosis infection in SCID mice. Microbes Infect. (2006) 8:1252–9. doi: 10.1016/j.micinf.2005.12.004 16702016

[64] Olivares N Rodriguez Y Zatarain-Barron ZL Marquina B Mata-Espinosa D Barrios-Payan J . A significant therapeutic effect of immunoglobulins administered alone, or in combination with conventional chemotherapy, in experimental pulmonary tuberculosis caused by drug-sensitive or drug-resistant strains. Pathog Dis. (2017) 75. doi: 10.1093/femspd/ftx118 29186408

[65] Zeng L Zuo Y Tang M Lei C Li H Ma X . A subunit vaccine Ag85A-LpqH focusing on humoral immunity provides substantial protection against tuberculosis in mice. iScience. (2025) 28:111568. doi: 10.1016/j.isci.2024.111568 39868033 PMC11760819

[66] Lu LL Chung AW Rosebrock TR Ghebremichael M Yu WH Grace PS . A functional role for antibodies in tuberculosis. Cell. (2016) 167:433–43:e414. doi: 10.1016/j.cell.2016.08.072 27667685 PMC5526202

[67] Grace PS Peters JM Sixsmith J Lu R Irvine EB Luedeman C . Antibody-Fab and -Fc features promote Mycobacterium tuberculosis restriction. Immunity. (2025) 58:1586–97:e1585. doi: 10.1016/j.immuni.2025.05.004 40449485 PMC12476823

[68] Zimmermann N Thormann V Hu B Kohler AB Imai-Matsushima A Locht C . Human isotype-dependent inhibitory antibody responses against Mycobacterium tuberculosis. EMBO Mol Med. (2016) 8:1325–39. doi: 10.15252/emmm.201606330 27729388 PMC5090662

[69] Ishida E Corrigan DT Chen T Liu Y Kim RS Song L . Mucosal and systemic antigen-specific antibody responses correlate with protection against active tuberculosis in nonhuman primates. EBioMedicine. (2024) 99:104897. doi: 10.1016/j.ebiom.2023.104897 38096687 PMC10758715

[70] Lu LL Das J Grace PS Fortune SM Restrepo BI Alter G . Antibody Fc glycosylation discriminates between latent and active tuberculosis. J Infect Dis. (2020) 222:2093–102. doi: 10.1093/infdis/jiz643 32060529 PMC7661770

[71] Melkie ST Arias L Farroni C Jankovic Makek M Goletti D Vilaplana C . The role of antibodies in tuberculosis diagnosis, prophylaxis and therapy: a review from the ESGMYC study group. Eur Respir Rev. (2022) 31(163):210218. doi: 10.1183/16000617.0218-2021 35264411 PMC9489037

[72] Irvine EB O'Neil A Darrah PA Shin S Choudhary A Li W . Robust IgM responses following intravenous vaccination with Bacille Calmette-Guerin associate with prevention of Mycobacterium tuberculosis infection in macaques. Nat Immunol. (2021) 22:1515–23. doi: 10.1038/s41590-021-01066-1 34811542 PMC8642241

[73] Watson A Li H Ma B Weiss R Bendayan D Abramovitz L . Human antibodies targeting a Mycobacterium transporter protein mediate protection against tuberculosis. Nat Commun. (2021) 12:602. doi: 10.1038/s41467-021-20930-0 33504803 PMC7840946

[74] Tran AC Diogo GR Paul MJ Copland A Hart P Mehta N . Mucosal therapy of multi-drug resistant tuberculosis with IgA and interferon-gamma. Front Immunol. (2020) 11:582833. doi: 10.3389/fimmu.2020.582833 33193394 PMC7606302

[75] Tran AC Diogo GR Paul MJ Copland A Hart P Mehta N . Mucosal therapy of multi-drug resistant tuberculosis with IgA and interferon-γ. Front Immunol. (2020) 11:582833. doi: 10.3389/fimmu.2020.582833 33193394 PMC7606302

[76] Fischinger S Cizmeci D Shin S Davies L Grace PS Sivro A . A Mycobacterium tuberculosis specific IgG3 signature of recurrent tuberculosis. Front Immunol. (2021) 12:729186. doi: 10.3389/fimmu.2021.729186 34630406 PMC8493041

[77] Liu Y Chen T Zhu Y Furey A Lowary TL Chan J . Features and protective efficacy of human mAbs targeting Mycobacterium tuberculosis arabinomannan. JCI Insight. (2023) 8(20):e167960. doi: 10.1172/jci.insight.167960 37733444 PMC10619501

[78] Wen Z Wang L Ma H Li L Wan L Shi L . Integrated single-cell transcriptome and T cell receptor profiling reveals defects of T cell exhaustion in pulmonary tuberculosis. J Infect. (2024) 88:106158. doi: 10.1016/j.jinf.2024.106158 38642678

[79] Qin Y Wang Q Shi J . Immune checkpoint modulating T cells and NK cells response to Mycobacterium tuberculosis infection. Microbiol Res. (2023) 273:127393. doi: 10.1016/j.micres.2023.127393 37182283

[80] Wang PH Wu MF Hsu CY Lin SY Chang YN Lee HS . The dynamic change of immune checkpoints and CD14+ monocytes in latent tuberculosis infection. Biomedicines. (2021) 9(10):1479. doi: 10.3390/biomedicines9101479 34680598 PMC8533229

[81] Kamboj D Gupta P Basil MV Mohan A Guleria R Bhatnagar A . Improved Mycobacterium tuberculosis clearance after the restoration of IFN-gamma(+) TNF-alpha(+) CD4(+) T cells: impact of PD-1 inhibition in active tuberculosis patients. Eur J Immunol. (2020) 50:736–47. doi: 10.1002/eji.201948283 32113187

[82] Kauffman KD Sakai S Lora NE Namasivayam S Baker PJ Kamenyeva O . PD-1 blockade exacerbates Mycobacterium tuberculosis infection in rhesus macaques. Sci Immunol. (2021) 6(55):eabf3861. doi: 10.1126/sciimmunol.abf3861 33452107 PMC8300572

[83] Ogishi M Yang R Aytekin C Langlais D Bourgey M Khan T . Inherited PD-1 deficiency underlies tuberculosis and autoimmunity in a child. Nat Med. (2021) 27:1646–54. doi: 10.1038/s41591-021-01388-5 34183838 PMC8446316

[84] Tezera LB Bielecka MK Ogongo P Walker NF Ellis M Garay-Baquero DJ . Anti-PD-1 immunotherapy leads to tuberculosis reactivation via dysregulation of TNF-alpha. Elife. (2020) 9:e52668. doi: 10.7554/eLife.52668 32091388 PMC7058383

[85] Lombardi A Villa S Castelli V Bandera A Gori A . T-cell exhaustion in Mycobacterium tuberculosis and nontuberculous mycobacteria infection: pathophysiology and therapeutic perspectives. Microorganisms. (2021) 9(12):2460. doi: 10.3390/microorganisms9122460 34946062 PMC8704935

[86] Kang J Wei ZF Li MX Wang JH . Modulatory effect of Tim-3/Galectin-9 axis on T-cell-mediated immunity in pulmonary tuberculosis. J Biosci. (2020) 45:60. doi: 10.1007/s12038-020-0023-z 32345786

[87] Huang Q Wu X Wang Z Chen X Wang L Lu Y . The primordial differentiation of tumor-specific memory CD8(+) T cells as bona fide responders to PD-1/PD-L1 blockade in draining lymph nodes. Cell. (2022) 185:4049–66:e4025. doi: 10.1016/j.cell.2022.09.020 36208623

[88] Piccaro G Aquino G Gigantino V Tirelli V Sanchez M Iorio E . Mycobacterium tuberculosis antigen 85B modifies BCG-induced antituberculosis immunity and favors pathogen survival. J Leukoc Biol. (2024) 115:1053–69. doi: 10.1093/jleuko/qiae014 38242866

[89] Headley CA Gautam S Olmo-Fontanez A Garcia-Vilanova A Dwivedi V Akhter A . Extracellular delivery of functional mitochondria rescues the dysfunction of CD4(+) T cells in aging. Adv Sci (Weinh). (2024) 11:e2303664. doi: 10.1002/advs.202303664 37990641 PMC10837346

[90] Headley CA Gautam S Olmo-Fontanez A Garcia-Vilanova A Dwivedi V Schami A . Mitochondrial transplantation promotes protective effector and memory CD4(+) T cell response during Mycobacterium tuberculosis infection and diminishes exhaustion and senescence in elderly CD4(+) T cells. Adv Sci (Weinh). (2024) 11:e2401077. doi: 10.1002/advs.202401077 39039808 PMC11423092

[91] Davies LRL Wang C Steigler P Bowman KA Fischinger S Hatherill M . Age and sex influence antibody profiles associated with tuberculosis progression. Nat Microbiol. (2024) 9:1513–25. doi: 10.1038/s41564-024-01678-x 38658786 PMC11153143

[92] Abil OZ Liu S Yeh YW Wu Y Sen Chaudhuri A Li NS . A mucosal vaccine formulation against tuberculosis by exploiting the adjuvant activity of S100A4-A damage-associated molecular pattern molecule. Vaccine. (2024) 42:126151. doi: 10.1016/j.vaccine.2024.07.052 39089961

[93] Mahendra A Haque A Prabakaran P Mackness BC Fuller TP Liu X . Honing-in antigen-specific cells during antibody discovery: a user-friendly process to mine a deeper repertoire. Commun Biol. (2022) 5:1157. doi: 10.1038/s42003-022-04129-7 36310321 PMC9618561

[94] Wardemann H Busse CE . Expression cloning of antibodies from single human B cells. Methods Mol Biol. (2019) 1956:105–25. doi: 10.1007/978-1-4939-9151-8_5 30779032

[95] Liu H Dass SA Wong MTJ Balakrishnan V Nordin F Tye GJ . Investigating the diagnostic and therapeutic potential of a T cell receptor (TCR)-like single domain antibody (sDAb)-human IgG1 antibody against heat shock protein (HSP) 16KDa/HLA-A2 for latent tuberculosis. Trop Med Infect Dis. (2024) 9(7):139. doi: 10.3390/tropicalmed9070139 39058181 PMC11281560

[96] An Y Ni R Zhuang L Yang L Ye Z Li L . Tuberculosis vaccines and therapeutic drug: challenges and future directions. Mol BioMed. (2025) 6:4. doi: 10.1186/s43556-024-00243-6 39841361 PMC11754781

[97] Dijkman K Aguilo N Boot C Hofman SO Sombroek CC Vervenne RAW . Pulmonary MTBVAC vaccination induces immune signatures previously correlated with prevention of tuberculosis infection. Cell Rep Med. (2021) 2:100187. doi: 10.1016/j.xcrm.2020.100187 33521701 PMC7817873

[98] Aguilo N Uranga S Marinova D Monzon M Badiola J Martin C . MTBVAC vaccine is safe, immunogenic and confers protective efficacy against Mycobacterium tuberculosis in newborn mice. Tuberculosis (Edinb). (2016) 96:71–4. doi: 10.1016/j.tube.2015.10.010 26786657 PMC4727503

[99] Scriba TJ Kaufmann SH Henri Lambert P Sanicas M Martin C Neyrolles O . Vaccination against tuberculosis with whole-cell mycobacterial vaccines. J Infect Dis. (2016) 214:659–64. doi: 10.1093/infdis/jiw228 27247343

[100] Ahmad F Umar MS Khan N Jamal F Gupta P Zubair S . Immunotherapy with 5, 15-DPP mediates macrophage M1 polarization and modulates subsequent Mycobacterium tuberculosis infectivity in rBCG30 immunized mice. Front Immunol. (2021) 12:706727. doi: 10.3389/fimmu.2021.706727 34777338 PMC8586420

[101] Hoft DF Blazevic A Selimovic A Turan A Tennant J Abate G . Safety and immunogenicity of the recombinant BCG vaccine AERAS-422 in healthy BCG-naive adults: A randomized, active-controlled, first-in-human phase 1 trial. EBioMedicine. (2016) 7:278–86. doi: 10.1016/j.ebiom.2016.04.010 27322481 PMC4909487

[102] Counoupas C Pinto R Nagalingam G Britton WJ Triccas JA . Protective efficacy of recombinant BCG over-expressing protective, stage-specific antigens of Mycobacterium tuberculosis. Vaccine. (2018) 36:2619–29. doi: 10.1016/j.vaccine.2018.03.066 29627232

[103] Figl J Kohler H Wedlich N Liebler-Tenorio EM Grode L Parzmair G . Safety and immunogenicity of recombinant Bacille Calmette-Guerin strain VPM1002 and its derivatives in a goat model. Int J Mol Sci. (2023) 24(6):5509. doi: 10.3390/ijms24065509 36982586 PMC10058566

[104] Dellagostin OA Borsuk S Oliveira TL Seixas FK . Auxotrophic Mycobacterium bovis BCG: Updates and perspectives. Vaccines (Basel). (2022) 10(5):802. doi: 10.3390/vaccines10050802 35632558 PMC9146772

[105] Saiga H Nieuwenhuizen N Gengenbacher M Koehler AB Schuerer S Moura-Alves P . The recombinant BCG DeltaureC::hly vaccine targets the AIM2 inflammasome to induce autophagy and inflammation. J Infect Dis. (2015) 211:1831–41. doi: 10.1093/infdis/jiu675 25505299

[106] Cotton MF Rabie H . Planning to introduce novel tuberculosis vaccines in high burden settings: How could this be done? Lancet Glob Health. (2023) 11:e484–5. doi: 10.1016/S2214-109X(23)00123-7 36925160 PMC10022833

[107] Martin C Marinova D Aguilo N Gonzalo-Asensio J . MTBVAC, a live TB vaccine poised to initiate efficacy trials 100 years after BCG. Vaccine. (2021) 39:7277–85. doi: 10.1016/j.vaccine.2021.06.049 34238608

[108] Levillain F Kim H Woong Kwon K Clark S Cia F Malaga W . Preclinical assessment of a new live attenuated Mycobacterium tuberculosis Beijing-based vaccine for tuberculosis. Vaccine. (2020) 38:1416–23. doi: 10.1016/j.vaccine.2019.11.085 31862194

[109] Wang C Lu J Du W Wang G Li X Shen X . Ag85b/ESAT6-CFP10 adjuvanted with aluminum/poly-IC effectively protects Guinea pigs from latent Mycobacterium tuberculosis infection. Vaccine. (2019) 37:4477–84. doi: 10.1016/j.vaccine.2019.06.078 31266673

[110] Ouaked N Demoitie MA Godfroid F Mortier MC Vanloubbeeck Y Temmerman ST . Non-clinical evaluation of local and systemic immunity induced by different vaccination strategies of the candidate tuberculosis vaccine M72/AS01. Tuberculosis (Edinb). (2023) 143:102425. doi: 10.1016/j.tube.2023.102425 38180028

[111] Bellini C Vergara E Bencs F Fodor K Bosze S Krivic D . Design and characterization of a multistage peptide-based vaccine platform to target Mycobacterium tuberculosis infection. Bioconjug Chem. (2023) 34:1738–53. doi: 10.1021/acs.bioconjchem.3c00273 37606258 PMC10587871

[112] Jenum S Tonby K Rueegg CS Ruhwald M Kristiansen MP Bang P . A phase I/II randomized trial of H56:IC31 vaccination and adjunctive cyclooxygenase-2-inhibitor treatment in tuberculosis patients. Nat Commun. (2021) 12:6774. doi: 10.1038/s41467-021-27029-6 34811370 PMC8608791

[113] Archer MC McCollum J Press C Dutill TS Liang H Fedor D . Stressed stability and protective efficacy of lead lyophilized formulations of ID93+GLA-SE tuberculosis vaccine. Heliyon. (2023) 9:e17325. doi: 10.1016/j.heliyon.2023.e17325 37366520 PMC10278894

[114] Tabarsi P Mamishi S Anjidani N Shahpari R Kafi H Fallah N . Comparative immunogenicity and safety of SpikoGen(R), a recombinant SARS-CoV-2 spike protein vaccine in children and young adults: An immuno-bridging clinical trial. Int Immunopharmacol. (2024) 127:111436. doi: 10.1016/j.intimp.2023.111436 38147778

[115] Lu J Guo X Wang C Du W Shen X Su C . Therapeutic effect of subunit vaccine AEC/BC02 on Mycobacterium tuberculosis post-chemotherapy relapse using a latent infection murine model. Vaccines (Basel). (2022) 10(5):825. doi: 10.3390/vaccines10050825 35632581 PMC9145927

[116] Wilson L Gracie L Kidy F Thomas GN Nirantharakumar K Greenfield S . Safety and efficacy of tuberculosis vaccine candidates in low- and middle-income countries: A systematic review of randomised controlled clinical trials. BMC Infect Dis. (2023) 23:120. doi: 10.1186/s12879-023-08092-4 36829123 PMC9951834

[117] de Lima MR Leandro A de Souza AL Barradas MM Roma EH Fernandes ATG . Safety and immunogenicity of an *in vivo* muscle electroporation delivery system for DNA-hsp65 tuberculosis vaccine in cynomolgus monkeys. Vaccines (Basel). (2023) 11(12):1863. doi: 10.3390/vaccines11121863 38140266 PMC10747856

[118] Wang N Liang Y Ma Q Mi J Xue Y Yang Y . Mechanisms of ag85a/b DNA vaccine conferred immunotherapy and recovery from Mycobacterium tuberculosis-induced injury. Immun Inflammation Dis. (2023) 11:e854. doi: 10.1002/iid3.854 37249284 PMC10187016

[119] Weng S Zhang J Ma H Zhou J Jia L Wan Y . B21 DNA vaccine expressing ag85b, rv2029c, and rv1738 confers a robust therapeutic effect against latent Mycobacterium tuberculosis infection. Front Immunol. (2022) 13:1025931. doi: 10.3389/fimmu.2022.1025931 36569899 PMC9768437

[120] Looney MM Hatherill M Musvosvi M Flynn J Kagina BM Frick M . Conference report: WHO meeting summary on mRNA-based tuberculosis vaccine development. Vaccine. (2023) 41:7060–6. doi: 10.1016/j.vaccine.2023.10.026 37872013

[121] Hu Z Lu SH Lowrie DB Fan XY . Research advances for virus-vectored tuberculosis vaccines and latest findings on tuberculosis vaccine development. Front Immunol. (2022) 13:895020. doi: 10.3389/fimmu.2022.895020 35812383 PMC9259874

[122] Wajja A Nassanga B Natukunda A Serubanja J Tumusiime J Akurut H . Optimising the vaccine strategy of BCG, ChAdOx1 85A, and MVA85A for tuberculosis control. Lancet Infect Dis. (2024) 24:e78–9. doi: 10.1016/S1473-3099(23)00758-2 38184003

[123] Jeyanathan M Vaseghi-Shanjani M Afkhami S Grondin JA Kang A D'Agostino MR . Parenteral BCG vaccine induces lung-resident memory macrophages and trained immunity via the gut-lung axis. Nat Immunol. (2022) 23:1687–702. doi: 10.1038/s41590-022-01354-4 36456739 PMC9747617

[124] Konjengbam BD Meitei HN Pandey A Haobam R . Goals and strategies in vaccine development against tuberculosis. Mol Immunol. (2025) 183:56–71. doi: 10.1016/j.molimm.2025.04.016 40327952

[125] Fan X Li X Wan K Zhao X Deng Y Chen Z . Construction and immunogenicity of a T cell epitope-based subunit vaccine candidate against Mycobacterium tuberculosis. Vaccine. (2021) 39:6860–5. doi: 10.1016/j.vaccine.2021.10.034 34702619

[126] Shi L Gu R Long J Duan G Yang H . Application of CRISPR-cas-based technology for the identification of tuberculosis, drug discovery and vaccine development. Mol Biol Rep. (2024) 51:466. doi: 10.1007/s11033-024-09424-6 38551745

[127] Zhou F Zhang D . Recent advance in the development of tuberculosis vaccines in clinical trials and virus-like particle-based vaccine candidates. Front Immunol. (2023) 14:1238649. doi: 10.3389/fimmu.2023.1238649 38022657 PMC10652786

[128] Nadeem S Maurya SK Das DK Khan N Agrewala JN . Gut dysbiosis thwarts the efficacy of vaccine against Mycobacterium tuberculosis. Front Immunol. (2020) 11:726. doi: 10.3389/fimmu.2020.00726 32508806 PMC7248201

[129] Morgun E Zhu J Almunif S Bobbala S Aguilar MS Wang J . Vaccination with mycobacterial lipid loaded nanoparticle leads to lipid antigen persistence and memory differentiation of antigen-specific T cells. Elife. (2023) 12:RP87431. doi: 10.7554/eLife.87431 37877801 PMC10599656

[130] Vergara EJ Tran AC Kim MY Mussa T Paul MJ Harrison T . Mucosal and systemic immune responses after a single intranasal dose of nanoparticle and spore-based subunit vaccines in mice with pre-existing lung mycobacterial immunity. Front Immunol. (2023) 14:1306449. doi: 10.3389/fimmu.2023.1306449 38130713 PMC10733481

[131] Fredsgaard-Jones T Harris SA Morrison H Ateere A Nassanga B Ramon RL . A dose escalation study to evaluate the safety of an aerosol BCG infection in previously BCG-vaccinated healthy human UK adults. Front Immunol. (2024) 15:1427371. doi: 10.3389/fimmu.2024.1427371 39611145 PMC11602284

[132] Ritter K Rousseau J Hölscher C . Interleukin-27 in tuberculosis: A sheep in wolf's clothing? Front Immunol. (2021) 12:810602. doi: 10.3389/fimmu.2021.810602 35116036 PMC8803639

[133] Arias AA Neehus AL Ogishi M Meynier V Krebs A Lazarov T . Tuberculosis in otherwise healthy adults with inherited TNF deficiency. Nature. (2024) 633:417–25. doi: 10.1038/s41586-024-07866-3 39198650 PMC11390478

[134] Sun M Phan JM Kieswetter NS Huang H Yu KKQ Smith MT . Specific CD4(+) T cell phenotypes associate with bacterial control in people who 'resist' infection with Mycobacterium tuberculosis. Nat Immunol. (2024) 25:1411–21. doi: 10.1038/s41590-024-01897-8 38997431 PMC11291275

[135] Shanmuganathan G Orujyan D Narinyan W Poladian N Dhama S Parthasarathy A . Role of interferons in Mycobacterium tuberculosis infection. Clin Pract. (2022) 12:788–96. doi: 10.3390/clinpract12050082 36286068 PMC9600403

[136] Zhu L Wang B Gu J Zhou J Wu Y Xu W . IFNγ-secreting T cells that highly express IL-2 potently inhibit the growth of intracellular M. tuberculosis in macrophages. Front Immunol. (2024) 15:1469118. doi: 10.3389/fimmu.2024.1469118 39575242 PMC11578947

[137] Mishra A Singh VK Actor JK Hunter RL Jagannath C Subbian S . GM-CSF dependent differential control of Mycobacterium tuberculosis infection in human and mouse macrophages: Is macrophage source of GM-CSF critical to tuberculosis immunity? Front Immunol. (2020) 11:1599. doi: 10.3389/fimmu.2020.01599 32793233 PMC7390890

[138] Hamilton F Schurz H Yates TA Gilchrist JJ Möller M Naranbhai V . Altered IL-6 signalling and risk of tuberculosis: A multi-ancestry mendelian randomisation study. Lancet Microbe. (2025) 6:100922. doi: 10.1016/s2666-5247(24)00162-9 39579785

[139] Khelghati F Rahmanian M Eghbal E Seghatoleslami ZS Goudarzi M Keramatinia A . Risk of tuberculosis disease in patients receiving TNF-α antagonist therapy: A meta-analysis of randomized controlled trials. New Microbes New Infect. (2024) 62:101533. doi: 10.1016/j.nmni.2024.101533 39639969 PMC11617757

[140] Mi J Liang Y Liang J Gong W Wang S Zhang J . The research progress in immunotherapy of tuberculosis. Front Cell Infect Microbiol. (2021) 11:763591. doi: 10.3389/fcimb.2021.763591 34869066 PMC8634162

[141] Ghoreschi K Balato A Enerbäck C Sabat R . Therapeutics targeting the IL-23 and IL-17 pathway in psoriasis. Lancet (London England). (2021) 397:754–66. doi: 10.1016/s0140-6736(21)00184-7 33515492

[142] Zhuo Q Zhang X Zhang K Chen C Huang Z Xu Y . The gut and lung microbiota in pulmonary tuberculosis: Susceptibility, function, and new insights into treatment. Expert Rev Anti-Infective Ther. (2023) 21:1355–64. doi: 10.1080/14787210.2023.2283036 37970631

[143] Han M Wang X Su L Pan S Liu N Li D . Intestinal microbiome dysbiosis increases Mycobacteria pulmonary colonization in mice by regulating the Nos2-associated pathways. Elife. (2024) 13:RP99282. doi: 10.7554/eLife.99282 39412514 PMC11483126

[144] Shah T Shah Z Baloch Z Cui X . The role of microbiota in respiratory health and diseases, particularly in tuberculosis. Biomedicine Pharmacotherapy = Biomedecine Pharmacotherapie. (2021) 143:112108. doi: 10.1016/j.biopha.2021.112108 34560539

[145] Arrigoni R Ballini A Topi S Bottalico L Jirillo E Santacroce L . Antibiotic resistance to Mycobacterium tuberculosis and potential use of natural and biological products as alternative anti-mycobacterial agents. Antibiotics (Basel Switzerland). (2022) 11(10):1431. doi: 10.3390/antibiotics11101431 36290089 PMC9598247

[146] Yang F Yang Y Chen L Zhang Z Liu L Zhang C . The gut microbiota mediates protective immunity against tuberculosis via modulation of lncRNA. Gut Microbes. (2022) 14:2029997. doi: 10.1080/19490976.2022.2029997 35343370 PMC8966992

[147] Pant A Das B Arimbasseri GA . Host microbiome in tuberculosis: Disease, treatment, and immunity perspectives. Front Microbiol. (2023) 14:1236348. doi: 10.3389/fmicb.2023.1236348 37808315 PMC10559974

[148] Shi W Hu Y Ning Z Xia F Wu M Hu YOO . Alterations of gut microbiota in patients with active pulmonary tuberculosis in China: A pilot study. Int J Infect Dis IJID Off Publ Int Soc For Infect Dis. (2021) 111:313–21. doi: 10.1016/j.ijid.2021.08.064 34481968

[149] Wang Y Deng Y Liu N Chen Y Jiang Y Teng Z . Alterations in the gut microbiome of individuals with tuberculosis of different disease states. Front Cell Infect Microbiol. (2022) 12:836987. doi: 10.3389/fcimb.2022.836987 35425720 PMC9001989

[150] Silva F Enaud R Creissen E Henao-Tamayo M Delhaes L Izzo A . Mouse subcutaneous BCG vaccination and Mycobacterium tuberculosis infection alter the lung and gut microbiota. Microbiol Spectr. (2022) 10:e0169321. doi: 10.1128/spectrum.01693-21 35652642 PMC9241886

[151] Bhattarai SK Du M Zeamer AL B MM Kellogg TD Firat K . Commensal antimicrobial resistance mediates microbiome resilience to antibiotic disruption. Sci Transl Med. (2024) 16:eadi9711. doi: 10.1126/scitranslmed.adi9711 38232140 PMC11017772

[152] Chai Y Liu X Bai G Zhou N Liu D Zhang X . Gut microbiome, T cell subsets, and cytokine analysis identify differential biomarkers in tuberculosis. Front Immunol. (2024) 15:1323723. doi: 10.3389/fimmu.2024.1323723 38650928 PMC11033455

[153] Chai Y Li M Deng X Ma C Zhou N Chen Y . Gut microbiota and tuberculosis infection: interaction and therapeutic potential. Gut Microbes. (2025) 17:2531201. doi: 10.1080/19490976.2025.2531201 40654283 PMC12269669

[154] Seo H Yoon Y Kim S Ghorbanian F Tajdozian H Jo S . Anti-tuberculosis effect of microbiome therapeutic PMC205 in extensively drug-resistant pulmonary tuberculosis *in vivo*. Int J Antimicrob Agents. (2024) 64:107274. doi: 10.1016/j.ijantimicag.2024.107274 39002701

[155] Jiang L Wang J Xu L Cai J Zhao S Ma A . Lactobacillus casei modulates inflammatory cytokines and metabolites during tuberculosis treatment: a post hoc randomized controlled trial. Asia Pac J Clin Nutr. (2022) 31:66–77. doi: 10.6133/apjcn.202203_31(1).0008 35357105

[156] Burrows K Ngai L Chiaranunt P Watt J Popple S Forde B . A gut commensal protozoan determines respiratory disease outcomes by shaping pulmonary immunity. Cell. (2024) 188(2):316–30.e12. doi: 10.1016/j.cell.2024.11.020 39706191 PMC11761380

[157] Strathdee SA Hatfull GF Mutalik VK Schooley RT . Phage therapy: from biological mechanisms to future directions. Cell. (2023) 186:17–31. doi: 10.1016/j.cell.2022.11.017 36608652 PMC9827498

[158] Advocating for phage therapy. Nat Microbiol. (2024) 9:1397–8. doi: 10.1038/s41564-024-01733-7 38839974

[159] Raman SK Siva Reddy DV Jain V Bajpai U Misra A Singh AK . Mycobacteriophages: therapeutic approach for mycobacterial infections. Drug Discov Today. (2024) 29:104049. doi: 10.1016/j.drudis.2024.104049 38830505

[160] Guerrero-Bustamante CA Dedrick RM Garlena RA Russell DA Hatfull GF . Toward a phage cocktail for tuberculosis: susceptibility and tuberculocidal action of mycobacteriophages against diverse Mycobacterium tuberculosis strains. mBio. (2021) 12(3):e00973–21. doi: 10.1128/mBio.00973-21 34016711 PMC8263002

[161] Yang F Labani-Motlagh A Bohorquez JA Moreira JD Ansari D Patel S . Bacteriophage therapy for the treatment of Mycobacterium tuberculosis infections in humanized mice. Commun Biol. (2024) 7:294. doi: 10.1038/s42003-024-06006-x 38461214 PMC10924958

[162] Singh AK Gangakhedkar R Thakur HS Raman SK Patil SA Jain V . Mycobacteriophage D29 Lysin B exhibits promising anti-mycobacterial activity against drug-resistant Mycobacterium tuberculosis. Microbiol Spectr. (2023) 11:e0459722. doi: 10.1128/spectrum.04597-22 37800970 PMC10714809

[163] Li X Xu J Wang Y Gomaa SE Zhao H Teng T . The biological characteristics of Mycobacterium phage Henu3 and the fitness cost associated with its resistant strains. Int J Mol Sci. (2024) 25(17):9310. doi: 10.3390/ijms25179301 39273250 PMC11394830

[164] Nayak T Kakkar A Jaiswal LK Kandwal G Singh AK Temple L . Characterization of a novel virulent mycobacteriophage Kashi-SSH1 (KSSH1) depicting genus-specific broad-spectrum anti-mycobacterial activity. Life Sci. (2025) 369:123546. doi: 10.1016/j.lfs.2025.123546 40058575

[165] Nayak T Kakkar A Singh RK Jaiswal LK Singh AK Temple L . Isolation and characterization of a novel mycobacteriophage Kashi-VT1 infecting Mycobacterium species. Front Cell Infect Microbiol. (2023) 13:1173894. doi: 10.3389/fcimb.2023.1173894 37545854 PMC10400892

[166] Wang X Su H Wallach JB Wagner JC Braunecker BJ Gardner M . Engineered Mycobacterium tuberculosis triple-kill-switch strain provides controlled tuberculosis infection in animal models. Nat Microbiol. (2025) 10:482–94. doi: 10.1038/s41564-024-01913-5 39794471 PMC11790485

[167] Dedrick RM Smith BE Cristinziano M Freeman KG Jacobs-Sera D Belessis Y . Phage therapy of Mycobacterium infections: compassionate use of phages in 20 patients with drug-resistant mycobacterial disease. Clin Infect Dis. (2023) 76:103–12. doi: 10.1093/cid/ciac453 35676823 PMC9825826

[168] Pathak V Chan HK Zhou QT . Formulation of bacteriophage for inhalation to treat multidrug-resistant pulmonary infections. Kona. (2025) 42:200–12. doi: 10.14356/kona.2025016 40114780 PMC11925536

[169] Gideon HP Hughes TK Tzouanas CN Wadsworth MH 2nd Tu AA Gierahn TM . Multimodal profiling of lung granulomas in macaques reveals cellular correlates of tuberculosis control. Immunity. (2022) 55:827–846.e810. doi: 10.1016/j.immuni.2022.04.004 35483355 PMC9122264

[170] Huang Z Gunarathne SMS Liu W Zhou Y Jiang Y Li S . PhIP-Seq: methods, applications and challenges. Front Bioinform. (2024) 4:1424202. doi: 10.3389/fbinf.2024.1424202 39295784 PMC11408297

[171] Gao S Yang Z Yu J Zhang F Tang S Pang Y . Mitophagy: a potential therapeutic target for tuberculosis immunotherapy. Immunotargets Ther. (2025) 14:773–86. doi: 10.2147/itt.S518628 40727489 PMC12301142

[172] Tailor NK Deswal G Guarve K Grewal AS . Development of Mycobacterium tuberculosis enoyl acyl reductase (InhA) inhibitors: a mini-review. Mini-Rev Med Chem. (2025) 25:219–33. doi: 10.2174/0113895575309785240902102421 39301902

[173] Goldberg K Lobov A Antonello P Shmueli MD Yakir I Weizman T . Cell-autonomous innate immunity by proteasome-derived defence peptides. Nature. (2025) 639:1032–41. doi: 10.1038/s41586-025-08615-w 40044870 PMC11946893

[174] Liu Q Que S Qiu Y Tang M Liu S Yang G . Host immune response to Mycobacterium tuberculosis infection: implications for vaccine development. J Inflammation Res. (2025) 18:8429–45. doi: 10.2147/jir.S517034 40599694 PMC12212080

[175] Fang R Yang X Li X Xing J Sun M Zhang Y . Host-directed immunotherapy to enhance treatment of Mycobacterium tuberculosis infection. Int Immunopharmacol. (2025) 165:115455. doi: 10.1016/j.intimp.2025.115455 40915191

[176] Mahieu L Van Moll L De Vooght L Delputte P Cos P . *In vitro* modelling of bacterial pneumonia: a comparative analysis of widely applied complex cell culture models. FEMS Microbiol Rev. (2024) 48(2):fuae007. doi: 10.1093/femsre/fuae007 38409952 PMC10913945

[177] Fröhlich E . Animals in respiratory research. Int J Mol Sci. (2024) 25(5):2903. doi: 10.3390/ijms25052903 38474149 PMC10931704

