# Supplementary material for: Immunotherapy for tuberculosis: emerging modalities, cross-disciplinary innovations, and roadmaps for drug-resistant disease
Source: Front Immunol. 2026 Jul 9;17:1873405. doi: 10.3389/fimmu.2026.1873405 (PMC13391292; doi:10.3389/fimmu.2026.1873405)
Supplement: Supplementary file 1 [file Table1.docx]

sTable1: Role of immune cell therapy in Tuberculosis Immunotherapy

| **Type** | **Mechanism** | **Research progress** | **Clinical trial stage** | **Challenges** |
| --- | --- | --- | --- | --- |
| LAK | LAK cells enhance the body's anti-infective capacity by activating and expanding NK cells and T cells in the immune system. | LAK cells derived from tuberculosis patients exhibit reduced ability to secrete cytokines such as IFN-γ. | Preclinical studies | 1. Non-targeted effects; 2. Use of IL-2 may induce severe immune-related adverse effects. |
| CIK | 1. CIK cells inhibit Mycobacterium tuberculosis (Mtb) by secreting cytokines (e.g., TNF-α, IFN-γ) and direct killing (e.g., releasing perforin, granzyme); 2. Synergy with factors such as IL-2 may amplify immune regulation and improve patient immune function. | CIK cell infusion can serve as an adjuvant for tuberculosis treatment, enhancing immune protection, improving chemotherapy efficacy, and reducing recurrence. | Phase I clinical trials (ChiCTR-INR-17012369) |  |
| γδ T | 1. Directly recognizes and kills Mtb-infected cells; 2. Secretes cytokines (e.g., IFN-γ, TNF-α) to directly kill pathogens and regulate other immune cells, enhancing host anti-infective capacity. | Clinical trials show good safety, with chest CT indicating significant reduction in lung lesions. | Phase I clinical trials (NCT05493267, NCT03575299) |  |
| NK | Participates in pathogen clearance via cytotoxicity and cytokine secretion. | In vitro-expanded NK cells effectively recognize and kill Mtb-infected cells and promote macrophage activation. | Preclinical studies |  |
| Adoptive T cell therapy (CD4+/CD8+) | 1. Activates macrophages to kill Mtb by secreting cytokines (e.g., IFN-γ, TNF-α); 2. Directly kills Mtb-infected macrophages. | In vitro expansion and reinfusion of Mtb-specific T cells enhance immune response. | Preclinical studies | 1. Complexity of Mtb antigens leads to T cell exhaustion; 2. Immunosuppressive microenvironment affects persistence. |
| DC | Promotes Th1-type immune responses by recognizing and presenting Mtb antigens. | Mtb antigen-pulsed DCs induce strong Th1-type immune responses and significantly reduce Mtb burden in lungs and spleens. | Phase I clinical trials (ChiCTR-INR-16009606) | 1. Insufficient screening of key antigens; 2. Safety requires verification. |
| CAR-T | Expresses specific antigen receptors to target and clear infected cells. | Generates Mtb-specific T cells from peripheral blood mononuclear cells via Rapid Expansion Protocol (REP) . | Preclinical studies | 1. Complex and variable Mtb antigens; no effective CAR-T cells yet; 2. Immunosuppressive microenvironment at infection sites may limit CAR-T function; 3. Safety: Cytokine storm. |

sTable 2: Research Progress of Costimulatory Molecules in Tuberculosis Immunotherapy

| **Molecule** | **Mechanism** | **Research progress** | **Clinical trial stage** | **Challenges** |
| --- | --- | --- | --- | --- |
| CTLA-4 | Inhibits CD28-mediated T cell activation to maintain immune tolerance. | Neutralizing CTLA-4 reduces Mtb counts in cell models. | Preclinical studies | 1. Balancing immune activation and suppression to avoid tissue damage; 2. Pleiotropic effects of costimulatory molecules across cell types; 3. Limitations of animal models and complexity of clinical trials; 4. Immune-related adverse effects and risk of TB recurrence; 5. High treatment costs and limited accessibility in resource-constrained regions. |
| PD-1/PD-L1 | Inhibits T cell function, leading to T cell exhaustion; high PD-1 expression correlates with disease progression in Mtb infection. | Results are conflicting: Significantly enhances CD4+ T cell proliferation and reduces Mtb burden in mouse tissues; however, PD-1 blockade in rhesus macaques exacerbates TB, and increased TB incidence is observed in cancer patients post-ICI therapy. | Phase I clinical trials (CTRI/2020/03/023815, ChiCTR2500095521) |  |
| ICOS | Promotes Th1/Th17 differentiation and memory T cell survival, enhancing anti-Mtb immune responses. | ICOS expression facilitates the generation of memory Mtb antigen-specific T cells. | Preclinical studies |  |
| 4-1BB (CD137) | Promotes IFN-γ secretion and cytotoxicity. | CD137 expression/signaling drives pleiotropic γδ T-cell effector functions that inhibit intracellular Mtb growth. | Preclinical studies |  |
| OX40 (CD134) | Enhances T cell survival and memory T cell formation to maintain long-term immune memory. | OX40 agonist immunotherapy decreases the lung bacterial burden and extends host survival. | Preclinical studies |  |
| CD40/CD40L | Boosts Th1 and Th17 immunity, expands memory CD4 T cell and CD8 T cell pools. | Reduction in the bacterial burden in the lungs was observed. | Preclinical studies |  |
| TIM-3 | Inhibits Th1/Tc1 cell function, induces T cell exhaustion, and maintains immune tolerance. | TIM-3 upregulation marks exhausted T cells in active TB; Blockade improves T-cell function in vitro. | Preclinical studies |  |
| CD30 | CD30 binds to its ligand CD153 to promote CD4+ T cell differentiation and protective effects. | In TB granulomas, CD30 signaling, provided by other T cells, promotes full differentiation of CD4+ T effector cells and enhances anti-TB immune responses. | Preclinical studies |  |
| LAG-3 | Inhibits T cell activation and proliferation, synergizes with PD-1 to induce T cell exhaustion. | Silencing LAG-3 signaling in macaque lung CD4+ T-cells enhanced killing of Mtb in co-cultures, accompanied by reduced mitochondrial electron transport and increased IFN-γ expression; the function of CD8+T cells was restored when LAG-3 signaling was blocked. | Preclinical studies |  |
| BTLA | Inhibits T cell activation and proliferation to maintain immune tolerance. | BTLA suppresses DC antigen presentation. | Preclinical studies |  |

sTable 3: Research Progress of Tuberculosis Vaccines

| **Type** | **Name** | **Design strategy** | **Purpose** | **Clinical trial stage** | **Mechanism** | **Challenges** |
| --- | --- | --- | --- | --- | --- | --- |
| Attenuated live vaccines | rBCG30 | Recombinant BCG | Prophylactic | Preclinical studies | Overexpresses Mtb antigen Ag85B to promote lymphocyte proliferation. | 1. Unclear immune response mechanisms, making it difficult to define ideal vaccine-induced immunity; 2. Unresolved mechanisms of immune exhaustion and dysregulation, limiting long-term memory; 3. Subunit vaccines rely on specific antigens (e.g., Ag85B, ESAT-6), but single antigens have limited coverage; require multi-antigen combinations or novel adjuvants; 4. Long trial cycles, high costs, and lack of standardized evaluation metrics. |
|  | AERAS-422 | Recombinant BCG | Prophylactic | Phase I（NCT01340820） | Overexpresses antigens Ag85A, Ag85B, and Rv3407; increases BCG antigen-specific IFN-γ secretion and macrophage counts. |  |
|  | VPM1002 | Recombinant BCG | Prophylactic | Phase III(NCT04351685,PACTR202007868402718) | Expresses Listeria lysin (Hly) and lacks urease C gene; stimulates Th17/Th1 responses and induces more central memory T cells (Tcm)、enhancing autophagy and inflammation. |  |
|  | MTBVAC | Attenuated Mtb strain | Prophylactic | Phase II/III(CTRI/2024/07/070928,NCT05947890,NCT04975178) | Lacks phoP and fadD26 genes，increases IL-1β expression, stimulates Th17/Th1 responses, and elevates immunoglobulin levels, being less reactogenic and more immunogenic than BCG. |  |
|  | GC1237 | Attenuated Mtb strain | Prophylactic | Preclinical studies | Double mutation in Rv1503c and PhoPR. |  |
| Inactivated vaccines | MIP | Heat-inactivated non-tuberculous mycobacteria | Therapeutic | Phase III(CTRI/2021/09/036335,CTRI/2024/06/068754 ) | Shares epitopes with Mycobacterium leprae and Mtb; induces Th1/Th17 immune responses, downregulates Th2 pathways, and activates macrophages/dendritic cells. |  |
|  | DAR-901 | Heat-inactivated non-tuberculous mycobacteria | Therapeutic/Prophylactic | Phase IIb(NCT02712424) | Shares epitopes with M. leprae and Mtb; significantly increases Mtb antigen-specific CD4+ IFN-γ, IL-2, and TNF-α, with cells displaying a polyfunctional effector memory phenotype. |  |
|  | RUTI | Liposome-encapsulated inactivated Mtb | Therapeutic | Phase IIb(NCT04919239) | Induces local accumulation of antigen-specific CD8+ T cells and strong humoral immune responses. |  |
|  | Tubivac (V7) | Heat-inactivated Mtb, oral formulation | Therapeutic | Phase III(NCT01977768) | Reduces TB-related inflammatory responses and increases white blood cell counts. |  |
| Recombinant protein subunit/adjuvant vaccines | GamTBvac | Antigens (ESAT-6, CFP-10, Ag85A) + adjuvant (CpG ODN) | Prophylactic | Phase III(NCT04975737) | Induced antigen-specific interferon-gamma release, Th1 cytokine-expressing CD4+ T-cells, and IgG responses. |  |
|  | M72/AC01E | Antigens (Mtb39A, Mtb32A) + adjuvant (AC01E) | Prophylactic | Phase III(NCT04975737) | Induces polyfunctional, Th1-cytokine-expressing M72-specific CD4+/CD8+ T cells in blood and lungs, with highest frequencies in lungs. |  |
|  | AEC/BC02 | Antigens (ESAT-6, CFP-10, Ag85B) + adjuvant (BC02) | Prophylactic | Phase II(NCT05284812  ) | Induces antigen-specific IFN-γ- or IL-2-secreting cellular immune responses; Ag85B- and EC-specific IgG observed in mice, indicating a significant Th1-biased response. |  |
|  | H4+IC31 | Antigens (TB10.4, Ag85B) + adjuvant (IC31) | Prophylactic | Phase II(PACTR201512001069358) | Induces CD4+ T cell response rates but no increase in H4-specific IgG binding antibodies. |  |
|  | H1+IC31 | Antigens (ESAT-6, Ag85B) + adjuvant (IC32) | Prophylactic/Therapeutic | Phase II(PACTR201105000289276) | Induces long-lived TNF-α+IL-2+ CD4 T cell responses. |  |
|  | ID93+GLA-SE | Antigens (Rv1813, Rv2608, Rv3619, Rv3620) + adjuvant (IC31) | Prophylactic/Therapeutic | Phase II(NCT06205589, NCT02465216, NCT03806686) | Exhibits significantly higher antigen-specific antibody levels and Type 1 T-helper cellular immune responses. |  |
|  | H56+IC31 | Antigens (ESAT-6, Rv2660, Ag85B) + adjuvant (IC31) | Therapeutic | Phase IIb(NCT03512249) | Induces robust expansion of antigen-specific T cells and a higher proportion of seroconversions. |  |
| Virus-like particle vaccines | ChAdOx1 85A | Recombinant adenovirus vaccine expressing TB antigen 85A (Ag85A) | Therapeutic | Phase I/II(NCT03681860) | Significantly increases Ag85A-specific T cells in vivo, which produce multiple cytokines (e.g., IFN-γ, TNF-α). |  |
|  | MVA85A | Modified vaccinia Ankara virus expressing Mtb antigen 85A | Prophylactic | Phase III(NCT00953927) | Induces significant CD4+ T cell responses and enhances BCG-induced immunity. |  |
|  | TB/FLU-04L | Attenuated influenza virus vector encoding Mtb antigens Ag85A and ESAT-6 | Prophylactic | Phase I(NCT02501421) | Induces lung-resident memory T cells and activates mucosal immunity. |  |
|  | Crucell Ad35+AERAS-402 | Adenovirus vector expressing Mtb antigens 85A, 85B, and TB10.4 | Prophylactic | Phase II(NCT01198366,NCT02414828,NCT01017536) | Induces strong polyfunctional CD4+ and CD8+ T cell responses. |  |
|  | EPER-VLP vaccine | Self-assembled via SpyCatcher-SpyTag technology | Therapeutic | Preclinical studies | Effectively triggers Th1 cellular immune responses and neutralizing antibody production. |  |
| Nucleic acid vaccines | DNA-hsp65 | DNA vector encoding Hsp65 gene | Therapeutic | Preclinical studies | Hsp65 gene is inserted into a plasmid vector and transfected into host cells, which express Hsp65 protein to activate CD4+/CD8+ T cell responses. |  |
|  | DNA-Ag85a/b | Ag85A or Ag85B gene inserted into a eukaryotic expression vector | Prophylactic/Therapeutic | Phase I(JPRN-jRCT2053190023) | Ag85A DNA vaccine significantly enhances anti-TB T cell responses and reduces bacterial burden in lungs/spleens of BCG-primed mice. |  |
|  | B21 DNA vaccine | Ag85B, Rv2029c, and Rv1738 | Therapeutic | Preclinical studies | Induces strong CD4+/CD8+ T cell activation and significantly increases secretion of cytokines (e.g., IFN-γ, TNF-α). |  |
|  | BNT164 | mRNA vaccine | Prophylactic | Phase I/II(NCT05547464,NCT05537038) | Induces potent T cell immunity. |  |
|  | AdHu5Ag85A | Recombinant human adenovirus type 5 (AdHu5) vaccine expressing Mtb Ag85A antigen | Prophylactic | Phase I(NCT02337270) | Significantly induces polyfunctional CD4+/CD8+ tissue-resident memory T cells in respiratory tissues. |  |

sTable 4: Research Progress of Cytokine in Tuberculosis Immunotherapy

| **Cytokine** | **Mechanism** | **Research progress** | **Clinical trial stage** | **Challenges** |
| --- | --- | --- | --- | --- |
| IL-1 | Pro-inflammatory cytokine, promotes immune response and inflammation. | Drives granuloma formation but excessive IL-1β correlates with tissue damage. β-glucan activates the IL-1 signaling pathway through epigenetic reprogramming, enhancing the host's innate immune defense against Mycobacterium tuberculosis (Mtb). IL-1 regulates eicosanoid metabolism to inhibit the overactivation of IFN-I, thereby limiting Mtb's immune escape. | Preclinical studies | 1. Safety. Overly excessive or uncontrolled inflammatory responses (such as those mediated by TNF-α, IL-1, IL-6, etc.) can lead to tissue damage, cavity formation, and disease exacerbation (immunopathological injury). On the other hand, immunosuppression (such as increased levels of TGF-β, IL-10) favors bacterial immune evasion and persistence. 2. Administration Routes and Targeting: Many cytokines with therapeutic potential (such as IFN-γ, IL-12) have short half-lives upon systemic administration, requiring frequent high-dose injections. This not only increases treatment costs and patient burden but also tends to cause severe systemic side effects. More importantly, how to specifically deliver cytokines to the infection site and target cells within tuberculous granulomas (such as macrophages, T cells), avoiding off-target effects on non-target tissues, is a bottleneck that current technology has not yet resolved. Local administration (such as inhalation) has been explored, but delivery efficiency and lesion penetration are still unsatisfactory. 3. Redundancy of the Immune Network and Individual Differences: The immune response is a complex network system composed of numerous cytokines, chemokines, and cells, with a high degree of redundancy. Intervention with a single or few cytokines may be compensated for or negated by other pathways, making it difficult to achieve the desired immunomodulatory effects. At the same time, host genetic background, nutritional status, co-infections (such as HIV), history of previous BCG vaccination, and differences in strains of Mycobacterium tuberculosis all result in significant individual differences in patients' responses to cytokine treatment, making prediction and standardization of treatment protocols extremely difficult. 4. Clinical Translation Barriers and Uncertainty of Efficacy: Preclinical research (especially in mouse models) results are unreliable in predicting human therapeutic effects. Many cytokine therapies that are effective in animal models (such as IFN-γ adjunctive therapy) have failed to demonstrate clear, consistent, and significant clinical benefits in human clinical trials (such as accelerating sputum conversion, shortening treatment duration, reducing relapse), with some research results even being contradictory. Well-designed, large-scale Phase III clinical trials are costly and time-consuming, and the results of existing Phase II studies are not yet sufficient to provide strong support. |
| IL-2 | T cell growth factor, promotes proliferation and differentiation of T cells. | IL-2 activates CD4+ T cells and regulates the balance of Th1/Th17/Treg to enhance anti-tuberculosis immune responses. Patients with tuberculosis often have suppressed IL-2 signaling pathways. Recombinant BCG-IL-2 enhances Th1 responses and CTL activity, while DNA vaccines (Hsp65-IL-2) reduce organ bacterial load. IL-2 has significant early efficacy in the diagnosis and treatment of pulmonary tuberculosis, with a significant increase in the 2-month sputum culture conversion rate (96.2% vs. 93.2%) and cavitation closure rate (28.4% vs. 18.5%), but there is no difference in long-term efficacy. In multidrug-resistant tuberculosis (MDR-TB), IL-2 has been shown to improve the cure rate, accelerate the conversion of sputum bacteria to negative and regulate the Th1/Th17/Treg balance. | Phase II/III, IV(NCT03069534, NCT04766307 ) |  |
| IL-3 | IL-3 is a hematopoietic growth factor that stimulates the proliferation and differentiation of bone marrow cells. It is involved in immune regulation and inflammatory responses. | IL-3 is a biomarker that can distinguish between a tuberculosis infection group and a healthy control group, and can differentiate between active and inactive tuberculosis. | Preclinical studies |  |
| IL-4 | Anti-inflammatory cytokine, promotes Th2 responses and antibody production. | IL-4 is associated with immunopathology in TB. High IL-4 levels may suppress protective Th1 responses. | Phase II(NCT 01638520) |  |
| IL-5 | Eosinophil activation and survival, involved in allergic responses. | IL-5 levels decrease in drug-resistant tuberculosis. IL-5 levels are higher in latent TB infection than in active tuberculosis. | Preclinical studies |  |
| IL-6 | Pro-inflammatory cytokine, regulates immune response and inflammation. | In patients with active tuberculosis (TB), elevated IL-6 damages effector T cell function. IL-6 significantly increases in the serum during the acute phase of active tuberculosis and decreases after treatment, serving as a marker for monitoring treatment effectiveness. Inhibition of IL-6 signaling may reduce the risk of tuberculosis. | Preclinical studies |  |
| IL-7 | T cell survival and homeostasis, promotes T cell proliferation. | Soluble IL-7R concentrations are reduced, and IL-7 concentrations are increased in tuberculosis patients. As a novel TB adjuvant, nonlytic Fc-fused IL-7 DNA (IL-7-nFc) may facilitate therapeutic TB DNA vaccines to the clinics. | Preclinical studies |  |
| IL-9 | Mainly produced by Th2 cells,involved in lymphocyte development and proliferation, enhance T cell survival and effector function. | A component of systemic immunity in LTBI and intradermal BCG, and pulmonary immunity following oral BCG. | Preclinical studies |  |
| IL-10 | Anti-inflammatory cytokines, inhibit Th1 cells from producing IFN-γ, but can enhance the secretion of IFN-γ by NK cells. | Interleukin-10-1082G/A polymorphism may play a significant role in the susceptibility to tuberculosis. IL10 inhibition can reduce the survival rate of intracellular pathogens Mycobacterium tuberculosis. | Preclinical studies |  |
| IL-12 | Pro-inflammatory cytokines, induce Th1 immune response, promote IFN-γ secretion. | Mutation in the IL-12Rβ1 gene associated with recurrent tuberculosis, discriminates between active TB disease and latent infection. | Preclinical studies |  |
| IL-15 | Stimulate the proliferation of NK cells and T cells, stimulate IFN-γ production. | rAd-IL-7-Linker-IL-15 improved the TB subunit vaccine's efficacy by augmenting TCM-like cells and provided long-term protective efficacy against Mycobacteria. | Preclinical studies |  |
| IL-17 | Regulating neutrophil recruitment and tissue inflammation. | IL-17A is significantly elevated in TB-PDM (Tuberculosis Pre-diabetes) patients, possibly aggravating the severity of the disease by enhancing inflammatory responses. | Preclinical studies |  |
| IL-18 | Pro-inflammatory cytokines, enhancing IFN-γ secretion. | Immune markers to differentiate between latent tuberculosis infection (LTBI) and active tuberculosis (ATB) . | Preclinical studies |  |
| IL-21 | Induces high-affinity antibody production from B cells, supports proliferation and effector functions of NK cells and CD8 T cells). | IL-21 as an adjuvant can enhance the immunogenicity of tuberculosis DNA vaccines. | Preclinical studies |  |
| IL-22 | Acts directly on epithelial cells to stimulate barrier function. | Higher in plasma samples from patients with tuberculosis, revaccination with BCG increased the frequency of CD4 T cell subsets expressing Th1 cytokines or IL-22. | Preclinical studies |  |
| IL-23 | Proinflammatory cytokine, maintains Th17 cell function. | p2AIL-23 is valuable as cytokine adjuvants for increasing the protective antituberculosis immunity induced by DNA vaccines. | Preclinical studies |  |
| IL-27 | Regulates Th1 and Th17 responses. | IL-27 is highly expressed in active tuberculosis, possibly affecting the immune response by regulating the Th1/Th2/Th17 balance. | Preclinical studies |  |
| IL-33 | Promotes Th2 responses, potentiates anti-tumor CD8+ T cells. | Immune markers to differentiate between latent tuberculosis infection (LTBI) and active tuberculosis (ATB) IL-33 may improve the immunogenicity of BCG vaccine by sensitizing hMCs systemic IL-33 treatment was effective in attenuating M. tuberculosis infection. | Preclinical studies |  |
| IL-35 | Inhibits the activation of monocyte-macrophage function and regulates the expression of various innate immune-related cytokines, thereby exerting immunosuppressive effects. | IL-35 gene polymorphism can be used to assess the severity of tuberculosis (PTB) disease and treatment response. | Preclinical studies |  |
| TNF-α | Pro-inflammatory cytokines involved in tumor necrosis, inflammatory response, and immune regulation. | double-edged sword, restricts the growth of mycobacteria within macrophages, maintains granulomas. Impaired T cell IFN-γ response mediated by suppression of TNF and non-canonical NF-κB signaling pathways might be responsible for disseminated tuberculosis. Both the TNF-α/IL-0 and TNF-α/TGF-β ratios may effectively categorize the tuberculosis severity and have the potential to serve as biomarkers for evaluating the early response to treatment. | Preclinical studies |  |
| TGF-β |  |  |  |  |
| IFN-α | IFN-α is produced by white blood cells and is primarily involved in innate immunity in response to viral infections. | Type I IFN-mediated NET release promotes Mycobacterium tuberculosis replication and is associated with granuloma caseation. | Preclinical studies |  |
| IFN-β | Stimulates the maturation of APCs, elevates costimulatory signals, and augments their capacity for antigen presentation or cross-presentation. | The death of Mtb-infected mouse macrophages in vitro is markedly exacerbated by induction of interferon-β (IFN-β). The Cyclooxygenase 2 Inhibitor Etoricoxib as Adjunctive Therapy in Tuberculosis reduces the production of macrophage IFN-β. | Preclinical studies |  |
| IFN-γ | Th1-type immune response cytokine, activates the bactericidal function of macrophages. | Interferon (IFN)-γ-producing CD8+ T cells contribute to control of Mtb infection, in part by promoting antimicrobial activities of macrophages. | Phase II(NCT05359315 NCT00001407 NCT00201123) |  |

| sTable 5: Research Progress of Microorganisms in Tuberculosis Immunotherapy | | | | |
| --- | --- | --- | --- | --- |
| **Type** | **Mechanism** | **Research progress** | **Current stage** | **Challenges** |
| Gut microbiota | Regulates CD4+/CD8+ T cells and inflammatory cytokines (IL-2, IL-4, IL-10) in the gut. | 1. Gut dysbiosis in TB patients (reduced Bacteroidetes, increased Proteobacteria)，fungal expansion and increased commensal bacterial abundance post-anti-TB treatment.  2. Gut microbiome impairs immunity in pulmonary TB patients via altered butyrate/propionate producers.  3. Bacteroides fragilis enhances lncRNA-CGB expression.  4. Supplementation with Lactobacillus casei significantly reduces inflammatory cytokines. | Phase II clinical trial (NCT03851159) | High individual variability requiring personalized intervention; strong strain specificity necessitating effective strain screening. |
| Lung microbiota | Maintains immune homeostasis in the lungs. | 1. Anti-TB treatment significantly affects alpha/beta diversity of lung microbiota. 2. Neisseria and Haemophilus in TB patients may influence Th1 immune responses. 3. Prevotella correlates positively with peripheral CD4+ cells in new TB patients but negatively in recurrent TB. 4. Helminths mediate immune tolerance to TB via gut microbiota. 5. Helicobacter hepaticus, associated with human biliary/liver diseases, alters gut microbiota and increases mouse susceptibility to Mtb. | Preclinical studies | Complex dynamics of lung microbiota; unclear causal relationships. |
| Mtb commensal bacteria | Regulates host immunity via metabolites (e.g., short-chain fatty acids). | Modulates Treg/Th1 balance to enhance anti-TB immunity. | Preclinical studies | Unclear mechanisms of commensal relationships; safety risks. |
| Microbiota metabolites (SCFAs) | Short-chain fatty acids (SCFAs) regulate gut microbiota and reduce adverse effects. | Improves anti-TB drug-induced gut adverse effects in rats by modulating gut microbiota and SCFAs. | Preclinical studies | Need to optimize metabolite stability and delivery methods. |
| Microbiota metabolites (itaconate) | Restricts growth of bacteria harboring isocitrate lyase. | Increased itaconate shows significantly attenuated virulence in a mouse low-dose aerosol infection model. | Preclinical studies |  |
| Microbiota metabolites (arginine) | L-arginine enhances autophagy and antimicrobial protection against Mtb in Kupffer cells. | Arginine promotes host anti-TB immunity. | Preclinical studies |  |
| D29 phage | Lysin A/B disrupts cell walls. | Effectively kills drug-sensitive and drug-resistant Mtb; synergizes with isoniazid. | Preclinical studies | Need to improve pulmonary targeted delivery (e.g., nebulization/nanocarriers); monitor phage-bacteria interactions dynamically; develop personalized cocktail regimens; current studies limited to animal models, requiring human trials. |
| DS6A phage | Efficiently lyses Mtb in liquid culture and macrophages. | Significantly reduces lung bacterial burden and improves lung function. | Preclinical studies |  |
| TM4 phage | Tail fiber proteins bind to mycobacterial surface receptors. | Penetrates macrophages to kill intracellular bacteria, reducing bacterial load by 90% and lung bacterial burden in animal models. | Preclinical studies |  |
| Phage cocktail therapy | Synergistic action of multiple phages. | Overcomes Mtb multidrug resistance. | Phase I clinical trials |  |
| Che12 phage | Specific adsorption followed by DNA injection and lysis. | Significantly prolongs survival of infected hosts and reduces tissue pathological damage. | Preclinical studies |  |
| Bacteriolytic phage KVT1 | Recombinant lysin (LysA) overexpression. | Penetrates necrotic cores to kill persister bacteria; synergizes with bedaquiline. | Preclinical studies |  |
| Clinical isolate phage | Phage-delivered antibiotic (clarithromycin). | No adverse effects observed across all patients, regardless of pathogen, phage type, or administration route; however, 4 patients showed poor efficacy. | Phase I clinical trials |  |
